# Wound, pressure ulcer, and burn guidelines (2023)―4: Guidelines for the management of connective tissue disease/vasculitis‐associated skin ulcers, third edition

**DOI:** 10.1111/1346-8138.17703

**Published:** 2025-04-28

**Authors:** Yoshihide Asano, Jun Asai, Takayuki Ishii, Yohei Iwata, Masanari Kodera, Chie Miyabe, Akihiko Uchiyama, Youichi Ogawa, Ken Okamura, Mari Kishibe, Yuta Koike, Yorihisa Kotobuki, Noriki Fujimoto, Takuya Miyagi, Yukie Yamaguchi, Ayumi Yoshizaki, Reiko Omori, Takeshi Nakanishi, Hiroshi Fujiwara, Takeo Maekawa, Sei‐ichiro Motegi, Yuichiro Yoshino, Minoru Hasegawa, Manabu Fujimoto, Takao Tachibana, Wound, Pressure Ulcer, and Burn Guidelines Drafting Committee (connective tissue disease/vasculitis group)

**Affiliations:** ^1^ Tohoku University; ^2^ Kyoto Prefectural University of Medicine; ^3^ Toyama Prefectural Central Hospital; ^4^ Fujita Health University; ^5^ Japan Community Health Care Organization (JCHO) Chukyo Hospital; ^6^ Tokyo Women's Medical University; ^7^ Gunma University; ^8^ University of Yamanashi; ^9^ Yamagata University; ^10^ Asahikawa Medical University; ^11^ Nagasaki University; ^12^ Osaka University; ^13^ Shiga University of Medical Science; ^14^ University of the Ryukyus; ^15^ Yokohama City University; ^16^ The University of Tokyo; ^17^ Meiji University of Integrative Medicine; ^18^ Niigata University; ^19^ Jichi Medical University Saitama Medical Center; ^20^ Japanese Red Cross Kumamoto Hospital; ^21^ University of Fukui; ^22^ Hoshigaoka Medical Center

**Table:** 

Chapter 1: Guidelines for the management of connective tissue disease/vasculitis‐associated skin ulcers (third edition)
Chapter 2: Outline of connective tissue disease/vasculitis‐associated skin ulcer diagnosis and treatment
Chapter 3: Guidelines for the management of connective tissue disease/vasculitis‐associated skin ulcers, CQs, and recommendations
CQ1: Are there any drugs effective in preventing or treating SSc‐associated skin ulcers?
CQ2: Are there any treatment methods effective for connective tissue disease–associated calcinosis cutis?
CQ3: Are there any drugs effective for vasculitis‐associated skin ulcers?
CQ4: Are there any treatment methods effective for rheumatoid vasculitis–associated skin ulcers?
Chapter 4: Definitions of terminology
Chapter 5: Explanation
Explanation 1: Effects of calcium antagonists, antiplatelet drugs, and prostaglandin preparations on SSc‐associated skin ulcers
Explanation 2: Surgical treatment for SSc‐associated skin ulcers
Explanation 3: Testing and treatment for SLE‐related skin ulcers and oral ulcers
Explanation 4: Examination and treatment for dermatomyositis‐associated skin ulcers
Explanation 5: Treatment for vasculitis‐associated skin ulcers
Explanation 6: Diagnosis and treatment of RA‐associated skin ulcers
Explanation 7: Diagnosis and treatment of antiphospholipid syndrome–associated skin ulcers
Chapter 6: Details of a systematic review on each CQ
List of the members of the Wound, Pressure Ulcer, and Burn Guidelines Drafting Committee
COI reporting criteria for participants in the Wound, Pressure Ulcer, and Burn Guidelines Supervising and Drafting Committees, participation/nonparticipation criteria, and a list of the COI disclosed

## 1 CHAPTER 1: GUIDELINES FOR THE MANAGEMENT OF CONNECTIVE TISSUE DISEASE/VASCULITIS‐ASSOCIATED SKIN ULCERS (THIRD EDITION)

### 1.1 Background to the drafting of guidelines for the management of connective tissue disease/vasculitis‐associated skin ulcers (third edition)

Guidelines are “documents systematically prepared to support medical experts and patients for making appropriate judgments in particular clinical situations.” Various clinical departments contribute to the management of connective tissue diseases and vasculitis, but dermatologists play a central role in evaluating skin lesions and treating skin ulcers. The Japanese Dermatological Association prepared these guidelines with an emphasis on the treatment of connective tissue disease– and vasculitis‐associated skin ulcers in the clinical setting of dermatology. Such ulcers occur against a background of various types of diseases, typically systemic sclerosis (SSc), but also include systemic lupus erythematosus (SLE), dermatomyositis, rheumatoid arthritis (RA), various vasculitides, and antiphospholipid antibody syndrome (APS). Therefore, in preparing the present guidelines, we considered the appropriate diagnostic/therapeutic approach for each of these disorders and developed corresponding algorithms and commentaries. The present guidelines aim to improve the quality and level of connective tissue disease– and vasculitis‐associated skin ulcer diagnosis/treatment in Japan by systematically presenting evidence‐based recommendations supporting clinical judgments.

### 1.2 Position of the guidelines for the management of connective tissue disease/vasculitis‐associated skin ulcers (third edition)

The Wound, Pressure Ulcer, and Burn Guidelines Drafting Committee was composed of members delegated by the Board of Directors of the Japanese Dermatological Association. It held several meetings and online meetings after the first meeting on June 3, 2018, and has drafted the explanations on wounds in general, and five other related guidelines, including these guidelines, by taking into consideration the opinions of the Scientific Committee and the Board of Directors of the Japanese Dermatological Association. The present guidelines reflect the current standards for diagnosis and treatment of skin ulcers in Japan. Background factors, such as the severity of symptoms and complications, vary among patients with connective tissue disease/vasculitis; therefore, physicians responsible for treatment should decide therapeutic strategies with patients. It is unlikely that the optimized treatment for an individual patient is in absolute agreement with these guidelines. Any deviation from these guidelines should not be the basis for citation in lawsuits or legal disputes. On the other hand, for treatment, the present situation in which treatment guidelines are quoted in lawsuits must be considered.

### 1.3 Major updated points in the third edition

- To improve transparency, we newly prepared guidelines according to the GRADE approach.
- Clinical question (CQ): A quantitative systematic review (meta‐analysis) and qualitative systematic review were performed with respect to four CQs that the Wound, Pressure Ulcer, and Burn Guidelines Drafting Committee members consider the most important.
- To ensure the convenience of the guidelines, an outline of the diagnosis and treatment of connective tissue disease– and vasculitis‐associated skin ulcers was divided into general remarks and detailed explanations with respect to diseases. A form to refer to a commentary with respect to the details of important points was adopted.

### 1.4 Sponsors

All expenses required for drafting these guidelines have been borne by the Japanese Dermatological Association, and no aid or financial support has been provided by organizations, enterprises or pharmaceutical companies.

### 1.5 Conflicts of interest

According to Japanese Association of Medical Sciences (JAMS) Guidelines on COI Management in Medical Research (https://jams.med.or.jp/guideline/index.html) published by JAMS in March 2017, the members of the guideline‐drafting committee disclosed the conflicts of interest (COI) during the past 3 years back to the previous year on assumption as a member and guideline announcement. For reporting: (1) the members' COI, their spouses' COI, (2) first‐degree relatives' or income/financial profit‐sharing persons' COI, and (3) COI of organizations/divisions to which the members belong were reported with an amount classification using the COI self‐disclosure form established in the JAMS Guidelines on COI Management in Medical Research.

### 1.6 Collection of evidence

The systematic review team for each CQ performed preliminary searching according to the Minds Manual for Clinical Practice Guideline Development 2020. The Japan Medical Library Association was responsible for searching.

- Databases used: PubMed, Cochrane Library, and Japanese Medical Abstracts Society
- Search period: Between January 1980 and the end of December 2020

### 1.7 Systematic review methods

According to the Minds Manual for Clinical Practice Guideline Development 2020, accompanying working templates were used.

#### 1.7.1 Evaluation of individual reports (step 1)

Systematic review teams responsible for individual CQs evaluated the bias risk (selection bias, performance bias, detection bias, patient attrition bias, other biases) and indirectness (differences in the study patient population/intervention/comparison/outcome measurement) of each study design (interventional study, observational study) with respect to the outcome‐based literature, and extracted the number of patients. When effect‐index‐presenting methods differed, they were unified to the risk ratio or risk difference, and described as total evidence.

#### 1.7.2 Summary on total evidence (step 2)

The total evidence integrated across outcomes was assessed with respect to a summary on total evidence, and the certainty of evidence was decided on one. The bias risk and indirectness were again evaluated. In addition, inconsistency, inaccuracy, and publication bias were assessed. The certainty (strength) of evidence was classified, as shown in Table 1.

**TABLE 1:** Classification of certainty (strength) of evidence.

A (strong)	There is strong confidence in the estimated value of effect
B (moderate)	There is moderate confidence in the estimated value of effect
C (weak)	Confidence in the estimated value of effect is limited
D (very weak)	We are not confident in the estimated value of effect

#### 1.7.3 Quantitative systematic review (meta‐analysis)

When the study design was similar, with the high‐degree similarity of each item of PICO (population, intervention, comparison, outcome), a meta‐analysis to quantitatively integrate effect indices was performed, and the integrated result was considered as an item for examining the strength of total evidence.

#### 1.7.4 Qualitative systematic review

When it was difficult to perform a quantitative systematic review (meta‐analysis), we performed a qualitative systematic review.

#### 1.7.5 Preparation of a systematic review report

The results of the above quantitative or qualitative systematic review were summarized in a systematic review report as the strength of total evidence, and used as materials for preparing recommendations with a summary on total evidence.

### 1.8 Recommendation‐determining methods

#### 1.8.1 Review by persons in charge of each CQ

We prepared a summary of findings, considering the certainty of total evidence regarding outcomes and balance between favorable (advantages) and adverse effects (harm and burden).

The importance (weighting) of favorable and adverse effects was re‐evaluated based on the importance of outcomes and certainty of total evidence. The direction and strength of recommendation were comprehensively considered, and submitted to a recommendation decision meeting through discussion by persons in charge of each CQ.

#### 1.8.2 Recommendation decision meeting

At a recommendation decision meeting (panel meeting), each systematic review team reported the results of examination based on the materials (evaluation sheets/total evidence, systematic review reports) submitted in advance. Subsequently, the members of the panel meeting voted for one of the following options:

- Recommended (strong recommendation)
- Proposed (weak recommendation)
- Not proposed (weak recommendation)
- Not recommended (strong recommendation)

For voting, the Delphi method was adopted. The recommendation level was determined with a consistency of ≥80%. When there was no consistency of ≥80% after three sessions of voting, the result was regarded as “no recommendation.”

Immediately before voting on each CQ, the presence or absence of a COI was reconfirmed, and panel meeting members with a COI did not vote. The results of voting are presented in the explanatory text for each CQ.

### 1.9 CQ changes in the process of preparation

There was no CQ change in the process of preparation.

### 1.10 Work for revising the guidelines

The Wound, Pressure Ulcer, and Burn Guidelines Drafting Committee held the First General Guideline Meeting on June 3, 2018, and started the revision. Subsequently, the supervising committee and six guideline‐drafting committees held online/mail meetings during the COVID‐19 pandemic. The committee for preparing the guidelines for the management of connective tissue disease/vasculitis‐associated skin ulcers (third edition) held an online recommendation decision meeting (panel meeting) on February 20, 2022, after a few sessions of mail meetings, and determined the recommendation level. Based on the results, each drafting committee member prepared a draft for the above guidelines, and the present guidelines were prepared through evaluation by the members of the Japanese Dermatological Association.

### 1.11 Committee for preparing the guidelines for the management of connective tissue disease/vasculitis‐associated skin ulcers (third edition)

Refer to the list of drafting committee members.

### 1.12 Review before publication

Before the publication of these guidelines, opinions were invited from the association members on the homepage of the Japanese Dermatological Association from 2022 to 2023, and necessary revisions were made.

### 1.13 Promotion of utilization after publication

The present guidelines will be announced at a general meeting of the Japanese Dermatological Association, and published in the *Japanese Journal of Dermatology*. Furthermore, anyone will be able to download them free of charge on the website of the Japanese Dermatological Association for widespread use. Furthermore, an English version of the guidelines will be published the year after publication.

**Committee for preparing the guidelines for the management of connective tissue disease/vasculitis‐associated skin ulcers (third edition)**

**Table:** 

	Name	Belonging to	Apportionment
Chairman of the supervising committee	Takao TACHIBANA	Department of Dermatology, Hoshigaoka Medical Center	Supervision
Vice‐chairman of the supervising committee	Minoru HASEGAWA	Department of Dermatology, University of Fukui	Supervision
Vice‐chairman of the supervising committee	Manabu FUJIMOTO	Department of Dermatology, Osaka University	Supervision
Supervising members	Takeshi NAKANISHI	Department of Dermatology, Meiji University of Integrative Medicine	Supervision
	Hiroshi FUJIWARA	Department of Dermatology, Niigata University	Supervision
	Takeo MAEKAWA	Department of Dermatology, Jichi Medical University Saitama Medical Center	Supervision
	Sei‐ichiro MOTEGI	Department of Dermatology, Gunma University	Supervision
	Yuichiro YOSHINO	Department of Dermatology, Japanese Red Cross Kumamoto Hospital	Supervision
Representative of the drafting committee	Yoshihide ASANO	Department of Dermatology, Tohoku University	Outline/CQ explanation writing, panel meeting
Drafting committee	Jun ASAI	Department of Dermatology, Kyoto Prefectural University of Medicine	Outline/CQ explanation writing, panel meeting
	Takayuki ISHII	Department of Dermatology, Toyama Prefectural Central Hospital	Outline/CQ explanation writing, panel meeting
	Yohei IWATA	Department of Dermatology, Fujita Health University	Outline/CQ explanation writing, panel meeting
	Masanari KODERA	Department of Dermatology, Japan Community Health care Organization (JCHO) Chukyo Hospital	Outline/CQ explanation writing, panel meeting
	Chie MIYABE	Department of Dermatology, Tokyo Women's Medical University	Outline/CQ explanation writing, panel meeting
	Reiko Omori	Nurse, The University of Tokyo	Panel meeting
**Systematic review team**		**Panel meeting members**	
CQ1	Akihiko UCHIYAMA, Youichi OGAWA, Yuta KOIKE	Jun ASAI, Takayuki ISHII, Yohei IWATA, Masanari KODERA, Chie MIYABE, Reiko Omori	
CQ2	Yorihisa KOTOBUKI, Takuya MIYAGI	Yoshihide ASANO, Jun ASAI, Takayuki ISHII, Yohei IWATA, Masanari KODERA, Chie MIYABE, Reiko Omori	
CQ3	Ken OKAMURA, Yukie YAMAGUCHI, Ayumi YOSHIZAKI	Yoshihide ASANO, Jun ASAI, Takayuki ISHII, Yohei IWATA, Masanari KODERA, Chie MIYABE, Reiko Omori	
CQ4	Mari KISHIBE, Noriki FUJIMOTO	Yoshihide ASANO, Jun ASAI, Takayuki ISHII, Yohei IWATA, Masanari KODERA, Chie MIYABE, Reiko Omori	

### 1.14 Plans for revision

The present guidelines are scheduled to be revised in the next 5 years. However, if a partial update becomes necessary, it will be presented on the website of the Japanese Dermatological Association when appropriate.

### 1.15 Monitoring after announcement

After guideline announcement, the widespread use of the present guidelines and changes in the contents of diagnosis/treatment will be investigated by a questionnaire survey.

### 1.16 Summary of CQs

**CQ1: Are there any drugs effective in preventing or treating SSc‐associated skin ulcers?**

**Prevention**

**Table:** 

Recommendation level	Description of recommendation
Bosentan: strong recommendation PDE5 inhibitors: weak recommendation	The prophylactic administration of bosentan for SSc‐associated skin ulcers is recommended. The prophylactic administration of PDE5 inhibitors (sildenafil, tadalafil) for SSc‐associated skin ulcers is proposed

**Treatment**

**Table:** 

Recommendation level	Description of recommendation
Bosentan: weak recommendation PDE5 inhibitors: weak recommendation	Treatment with bosentan or PDE5 inhibitors (sildenafil, tadalafil) for SSc‐associated skin ulcers is proposed

**CQ2: Are there any treatment methods effective for connective tissue disease–associated calcinosis cutis?**

**Table:** 

Recommendation level	Description of recommendation
Weak recommendation for all treatments	Treatment with warfarin, diltiazem hydrochloride, colchicine, or bisphosphonate preparations or by surgical resection for connective tissue disease–related calcinosis cutis is proposed. Treatment with rituximab for SSc‐related calcinosis cutis is proposed. It is proposed to avoid treatment with rituximab for dermatomyositis‐related calcinosis cutis

**CQ3: Are there any drugs effective for vasculitis‐associated skin ulcers?**

**Table:** 

Recommendation level	Description of recommendation
Weak recommendation	Systemic steroid administration is proposed because steroids are routinely used as a base drug for vasculitis‐associated skin ulcers in clinical practice, and because their effects have been obtained

**CQ4: Are there any treatment methods effective for rheumatoid vasculitis–associated skin ulcers?**

**Table:** 

Recommendation level	Description of recommendation
Weak recommendation for all drugs	Treatment with azathioprine in combination with steroids or alone, cyclophosphamide + steroid pulse, TNF‐α inhibitors, rituximab, or LCAP (leukocytapheresis)/GCAP (granulocyte and monocyte adsorption apheresis) for rheumatoid vasculitis–related skin ulcers is proposed

## 2 CHAPTER 2: OUTLINE OF CONNECTIVE TISSUE DISEASE/VASCULITIS‐ASSOCIATED SKIN ULCER DIAGNOSIS AND TREATMENT

### 2.1 General remarks

#### 2.1.1 Etiological factors for skin ulcers

Various diseases are included in collagen disease/vasculitis that forms skin ulcers, and their causes are diverse. However, some causes, such as circulatory impairment, infection, thrombosis, vasculitis, panniculitis, and calcinosis, are common to each of these diseases. Indeed, skin ulcers are not necessarily caused by one of these disorders alone. Caution is required because multiple factors, such as circulatory impairment plus infection or circulatory impairment plus thrombosis, may be involved. Resolution or elimination of these causative factors is indispensable for controlling skin ulcers. A brief overview of the management of each factor is presented below.

For circulatory disorders, the administration of oral drugs, such as beraprost sodium and sarpogrelate, and intravenous drugs, such as lipoprostaglandin E1 (lipo‐PGE1) preparations and argatroban hydrate, is evaluated. For fingertip ulcers due to SSc, warming in a kotatsu (heated table covered by a quilt) is also effective.

For infection, the systemic administration of antibacterial drugs is desirable when the “five signs of infection,” namely redness, swelling, heat, pain, and decreased function, are observed. Concerning topical agents, silver sulfadiazine cream or cadexomer iodine ointment is often used to remove necrotic tissue that has developed due to infection and treat the infection. However, the use of antibacterial drugs on the basis only of the detection of bacteria by culturing of wound samples should be avoided if clinical signs of infection are lacking. The indication for antibacterial drugs should be evaluated by judging whether the condition is due to colonization or infection. At the same time, ulcers due to connective tissue diseases often recur at the same sites, and the detection of infections of scarred wounds is likely delayed. Also, in patients with connective tissue diseases and vasculitis, as corticosteroids (steroids) and immunosuppressants are frequently used to treat the primary disease, attention to increased susceptibility to infection is necessary. Dressing materials are useful when the amount of exudate is high but are inappropriate when the wound is infected, as the ulcer may be exacerbated within a few days between dressing changes.

For thrombosis, the administration of anticoagulants, such as warfarin and various antiplatelet drugs, is necessary. As mentioned above, because thrombi may develop in stagnant blood due to circulatory disturbances or SLE may be concurrent with APS, the possibility of the involvement of multiple causative factors must be considered.

In vasculitis and panniculitis, necrosis of the cutaneous or subcutaneous tissue may occur in active lesions, leading to ulcer formation, or the ulceration of old scarred lesions may be triggered by the infection. For treating active vasculitis‐associated skin ulcers, the priority is to control the underlying disease primarily using steroids or immunosuppressants. However, if the skin ulcer is complicated by infection, intensification of the steroid or immunosuppressant therapy may exacerbate the ulcer due to the patient's increased susceptibility to infection. If there are signs of infection, its treatment must be performed simultaneously.

In panniculitis, judging whether the clinical symptoms, such induration and redness/heat, are caused by connective tissue diseases or infection is often difficult. Because blood test data indicate an elevated inflammatory reaction in either case, early differentiation is difficult. The examination of procalcitonin by blood sampling is also useful for confirming infection status because its levels often do not rise even in cases of high connective tissue disease activity. However, attention is required for the possibility of a false‐negative result caused by a nonspecific reaction in patients positive for rheumatoid factors and the fact that insurance coverage for procalcitonin measurement is limited to sepsis alone. Although a histopathological examination should be actively considered, it takes time to complete, and antibacterial drugs are often administrated as a diagnostic treatment in the actual clinical setting.

Calcium deposits also often cause ulcer formation if they spontaneously collapse. Regarding calcinosis, oral therapies, such as warfarin therapy, may be considered for treating small lesions, but large calcified lesions are not generally resolved by internal treatment alone and require surgical resection. However, as calcified lesions causing ulceration may extend broadly and occasionally deeply, early resection while the calcium deposits are still small may also be considered depending on treatment invasiveness if the patient responds poorly to internal treatment.

#### 2.1.2 Treatment with topical agents for skin ulcers

In treating skin ulcers, the selection of external drugs is an important factor. Silver sulfadiazine cream, dextranomer polymer, cadexomer iodine ointment, iodine ointment, povidone iodine sugar, a trafermin (basic fibroblast growth factor) preparation, tretinoin tocopherol ointment, bucladesine sodium ointment, PGE1 (alprostadil alfadex) ointment, and bromelain ointment are possible choices. The selection of these drugs should be made using wound bed preparation according to the tissue, infection, moisture, and wound edge (TIME) concept for treating pressure ulcers. Alternately, topical agents used for moist wound healing may also be useful for treating connective tissue disease/vasculitis‐associated skin ulcers.

Selection of topical agents for wound bed preparation.
T: Elimination of necrotic tissue: cadexomer iodine ointment.
I: Control/elimination of infection: cadexomer iodine ointment, silver sulfadiazine cream, povidone iodine sugar.
M: Maintaining a moist environment (control/elimination of exudates):
  when exudates are excessive, cadexomer iodine ointment, dextranomer polymer, povidone iodine sugar;
  when exudates are deficient, silver sulfadiazine cream.
E: Management of wound margins (resolution/elimination of pockets): no drugs recommended.
Selection of topical agents for moist wound healing.
  Wound surfaces with appropriate/deficient exudates: trafermin spray, PGE1 ointment.
  Wound surfaces with deficient exudates: tretinoin tocopherol ointment.
  Wound surfaces with excessive exudates or marked edema: bucladesine sodium ointment.

However, because patients with connective tissue disease/vasculitis‐associated skin ulcers often complain of intense pain, the use of topical agents according to these principles is occasionally impossible in cases in which topical agents cause irritating symptoms. The simple protection of the wound using white petrolatum or a petrolatum‐based ointment is selected in such cases.

#### 2.1.3 Treatment with dressing materials for skin ulcers

In recent years, occlusive dressings are frequently employed for treating ulcers due to improvements in dressing materials, but a cautious attitude is urged for their use for treating connective tissue disease–associated ulcers. Moist wound healing using an occlusive dressing is expected when wound bed preparation has been achieved and the ulcer condition is improving. However, with connective tissue disease–related ulcers, the condition may change readily in a short period and the ulcers can become exacerbated rapidly over a few days between dressing changes, thus making them worse rather than better. There have been instances of rapid exacerbation in a short period even of improving ulcers in cases of unstable control of the circulatory condition or underlying disease, as well as when the ulcer is infected, as mentioned above. There are also intractable ulcers not expected to heal except by spontaneous separation due to dry gangrene, and an occlusive dressing is inappropriate in such wounds.

#### 2.1.4 Surgical treatment for skin ulcers

Some comments about surgical treatments should also be made. Connective tissue disease–associated ulcers differ from other ulcers in that their condition changes readily depending on primary disease activity. For example, in fingertip ulcers in patients with SSc, wound bed preparation can be achieved by relieving circulatory disturbances and ulcers may be controlled by conservative treatment. On the other hand, circulatory disorders may recur readily at sites of resolved ulcers, resulting in their repeated reactivation. Considering these pathologies, as aggressive surgical treatments, such as finger/toe amputation, may lead to more extensive successive amputations, their indications must be evaluated carefully. Because tissues on the verge of necrosis may be saved by patient repetition of conservative treatments, maximum tissue conservation should also be achieved using debridement unless the tissue is clearly gangrenous. For bedside wound treatment, although the removal of marked necrotic tissue is necessary, the ischemic basement tissue should be preserved whenever possible. Removing marked necrotic tissue without injuring the ischemic tissue of a skin ulcer with forceps can reduce patient pain during wound treatment. When treating connective tissue disease/vasculitis‐associated skin ulcers, the patient's unique progress must be considered. Priority should be given to persistent conservative treatments, with surgical therapy progressing from skin grafting to bone curettage (exposure of bone marrow), and, if necessary, to finger/toe amputation, adhering to the principle of conservation or minimally invasive treatment. As a general rule, surgical therapy should be indicated on the condition that the primary disease is adequately controlled, with the exception of infections that require urgent treatment such as necrotizing fasciitis and gas gangrene.

#### 2.1.5 Pain control for skin ulcers

Pain management can also be an issue for connective tissue disease/vasculitis‐associated skin ulcers. Acute pain symptoms are often controlled using nonsteroidal anti‐inflammatory drugs or acetaminophen, but caution is needed to prevent kidney and liver function disorders. For intractable pain symptoms, tramadol hydrochloride/acetaminophen, tramadol hydrochloride, and codeine phosphate are the opioids used to manage mild to moderate pain. For patients with highly active conditions and infected skin ulcers, there are also rare cases in which morphine hydrochloride or fentanyl patches (with the requirement of e‐learning course completion for their appropriate use) are used as opioids for moderate to intense pain. There are also cases in which inflammation in the blood vessels that nourish the peripheral nerves can cause peripheral neuropathic pain where analgesia using pregabalin, mirogabalin, or duloxetine is effective. A nerve block can also be useful in certain patients.

It is the role of the dermatologist to infer the patient's general condition from skin symptoms, understand the patient's condition through various tests, and perform treatments accordingly. It is our hope that the present guidelines will be useful in the clinical setting.

### 2.2 Detailed explanation

#### 2.2.1 SSc‐associated skin ulcers

SSc exhibits fibrosis of the skin and various organs and vasculopathy as primary manifestations and is a frequent cause of skin ulcers/gangrene in connective tissue diseases. Existing ulcer/gangrene and resulting functional impairments markedly affect patient quality of life (QOL). In patients with SSc, ulcers often develop at the finger/toe tips resulting from peripheral circulatory insufficiency. They are likely to occur on the dorsal aspects of the finger joints accompanying skin sclerosis and flexion contractures. They are also frequently observed at the heel and internal and external malleoli. While ulcers at the finger/toe tips occur more frequently in winter, they are perennial in some patients. Gangrene may develop suddenly. It is not uncommon for small injuries to develop into intractable ulcers, and surgical wounds are no exception. Many patients judged to have sufficient preoperative blood flow experience ulceration of surgical wounds. Ulcers developing due to spontaneous collapse of subcutaneous calcium deposits and those due to infection of corns or their inappropriate treatment (particularly self‐treatment) are often encountered. SSc may be complicated by other connective tissue diseases/vasculitis, and attention to APS is necessary. Moreover, as even patients who do not fulfill the diagnostic criteria of SSc (e.g. those who are positive for anticentromere antibody and exhibit Reynaud phenomenon but have no finger/toe sclerosis) may develop ulcers/gangrene, they should be managed without excessive devotion to the diagnostic criteria.

For treating ulcers in SSc patients, it is necessary to keep the affected areas stationary and warm and attempt various combinations of topical and systemic drug therapies while eliminating endogenous and exogenous exacerbating factors. In surgical treatment, because local procedures, such as excessive debridement may enlarge ulcers, sufficient caution is needed. Similarly, finger/toe amputation for gangrene often invites further proximal extension of the wound from the stump. It is extremely important to give priority to conservative treatments and avoid unnecessary surgical stress when treating ulcers/gangrenes of SSc, and waiting for drying and spontaneous separation (autoamputation) of gangrenous tissue is often recommended.

While few individual systemic drug therapies have been shown to be useful for treating ulcers alone, this does not imply that they lack utility. Clinical experience shows that combinations of multiple drugs can be effective. This is also the case with topical treatments, and drugs must be selected according to wound condition. PGE1 ointments and trafermin sprays are frequently used.

Avoiding cold and encouraging rest are also important. In some patients, rapid remission is achieved by changing outpatient care to inpatient care. Pain control of skin ulcers is also important.

**FIGURE 1:**
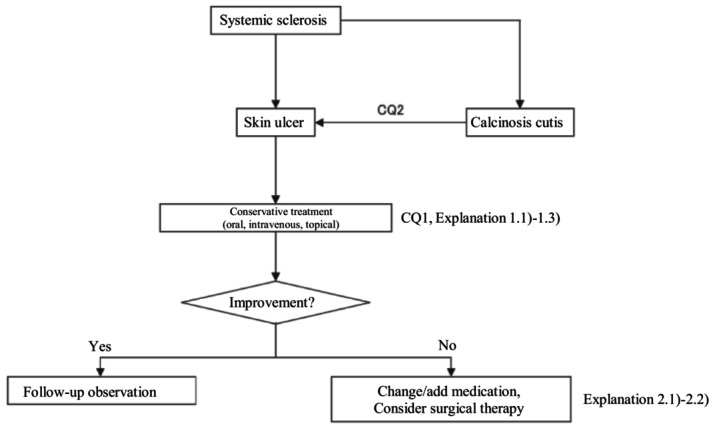
Therapeutic algorithm for systemic sclerosis–associated skin ulcers. CQ, clinical question.

Based on the above concept, an algorithm for SSc‐associated skin ulcers (Figure 1) was prepared, and the details are described in CQ1, CQ2, Explanation 1, and Explanation 2.

#### 2.2.2 SLE‐associated skin ulcers

SLE exhibits various rashes and occasionally erosion or ulcers. Representative skin manifestations of SLE are as follows: among the diagnostic criteria in Japan are the butterfly rash (malar rash), discoid LE, oral ulcers, and photosensitivity; while those not among the diagnostic criteria include chilblain LE, LE profundus, nodular cutaneous lupus mucinosis, bullous LE, and lupus tumidus. SLE rashes alone are rarely an indication for systemic administration of a steroid or an immunosuppressant and are treated topically. Oral treatment with hydroxychloroquine may also be used. As an exception, the early systemic administration of a steroid is indicated for LE profundus because of the possibility of subsequent scarring or pit formation. Nonspecific skin symptoms observed in SLE include those due to peripheral vascular or circulatory disorders, and skin ulcers or gangrene may appear. Skin biopsy and various blood tests are necessary for their differentiation from exanthema due to APS or vasculitis. Circulation‐improving drugs and antiplatelet agents are administrated to treat peripheral vascular and circulatory disorders as needed. If APS or vasculitis has been diagnosed, treatment for these conditions becomes necessary. For details, see the relevant sections of the present guidelines or other guidelines of the Japanese Dermatological Association. In addition, because such patients are often immunocompromised, ulcers can frequently be caused by various infectious diseases, and cases of squamous cell carcinoma forming from scars at intractable ulcers found in discoid LE have been reported.

**FIGURE 2:**
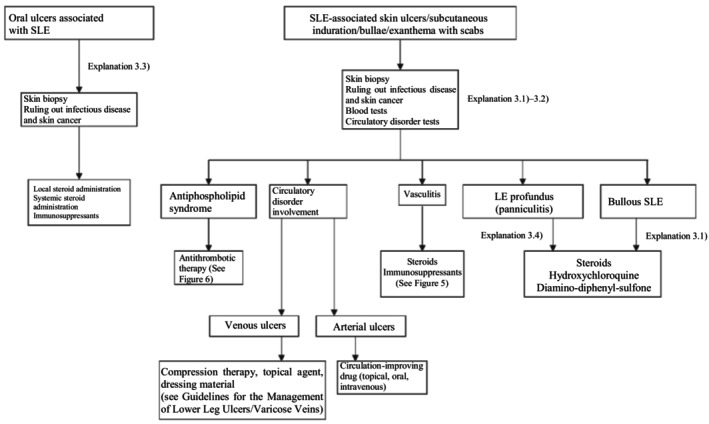
Therapeutic algorithm for systemic lupus erythematosus (SLE)–associated skin ulcers.

Based on the above concept, an algorithm for SLE‐associated skin ulcers and oral ulcers (Figure 2) was prepared, and the details are described in Explanation 3.

#### 2.2.3 Dermatomyositis‐associated skin ulcers

Dermatomyositis exhibits various rashes as well as erosion or ulcers. Common cutaneous manifestations occurring with dermatomyositis include Gottron papule (papules affecting the dorsal surfaces of finger joints), Gottron sign, (“keratosis” erythema affecting the dorsal surfaces of limb joints and finger joints), mechanic's hand (keratosis of the ulnar side of the thumb and the radial side of the index/middle fingers), heliotrope rash (reddish purple edematous erythema around the eyelids), facial erythema or edema, scratch dermatitis, poikiloderma, periungual erythema, nailfold bleeding, skin ulcers, calcinosis, and bullae. Causes of erosion and ulcers in dermatomyositis are diverse and include undermining ulcers with necrosis and purpura accompanying angiopathy, shallow secondary skin ulcers or erosion accompanying severe scratch dermatitis, calcinosis cutis, and panniculitis. Treating according to the cause is important.

In recent years, new and highly disease‐specific autoantibodies are being discovered for dermatomyositis, and their associations with clinical features have been clarified. Dermatomyositis in which anti‐melanoma differentiation‐associated gene 5 (anti‐MDA5) antibodies are detected presents as clinically amyopathic dermatomyositis (CADM), in which skin symptoms are typical but there is no clear myopathy, and it is known to accompany rapidly progressing interstitial lung disease at a high incidence. Skin symptoms may include undermining ulcers with purpura and necrosis. On the other hand, dermatomyositis in which anti‐transcription intermediary factor 1γ (anti–TIF‐1γ) antibodies are detected is known to be associated with a high rate of complications by visceral malignancy. Widespread severe skin inflammation is characteristic, and scratch dermatitis, bullae, erosion, and shallow ulcers often form. Histopathologically, pronounced infiltration of the dermoepidermal junction by inflammatory cells is characteristic. Thus, in the diagnosis and treatment of dermatomyositis‐associated ulcers, it is important to fully investigate/evaluate the disease condition according to the form and distribution of ulcers and other cutaneous manifestations. This should also include important complications such as rapidly progressing interstitial lung disease or visceral malignancy. The causes of skin ulcers do not necessarily align with the disease condition of dermatomyositis; thus, ulcer treatment should be considered separate from systemic treatment.

**FIGURE 3:**
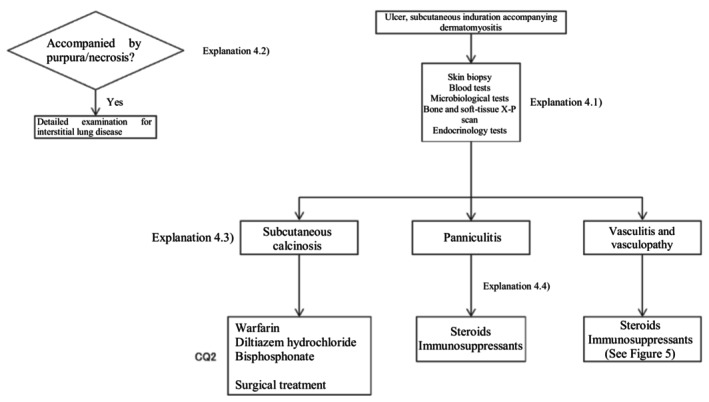
Therapeutic algorithm for dermatomyositis‐associated skin ulcers. CQ, clinical question.

Based on the above concept, an algorithm for dermatomyositis‐associated skin ulcers (Figure 3) was prepared, and the details are described in CQ4 and Explanation 4.

#### 2.2.4 Vasculitis‐associated skin ulcers

Vasculitis, a group of diseases caused primarily by necrotizing vasculitis observed histopathologically, is often a direct cause of skin ulcers. It is described by the Chapel Hill classification issued in 1994 (commonly called “CHCC 1994”), which was revised in 2012 (commonly called “CHCC 2012,”^1^ Table 2). In addition to the traditional three size‐based categories of large‐, medium‐, and small‐vessel vasculitis, the CHCC 2012 includes a total of seven categories by the addition of variable vessel vasculitis, single‐organ vasculitis, vasculitis associated with systemic disease, and vasculitis associated with probable etiology.

**TABLE 2:** Types of vasculitis in the Chapel Hill classification (2012) that commonly cause lesions such as skin ulcers.

Small‐vessel vasculitis
Microscopic polyangiitis
Eosinophilic granulomatosis with polyangiitis
(previous name: Churg–Strauss)
Granulomatosis with polyangiitis (previous name: Wegener's)
Immunoglobulin A vasculitis (previous name: Henoch‐Schönlein)
Cryoglobulinemic vasculitis
Hypocomplementemic urticarial vasculitis
Medium‐vessel vasculitis
Polyarteritis nodosa
Large‐vessel vasculitis
Giant cell arteritis
Variable vessel vasculitis
Behçet disease
Single‐organ vasculitis
Cutaneous leukocytoclastic angiitis
Cutaneous arteritis (previous name: cutaneous polyarteritis nodosa)
Vasculitis associated with systemic disease
Lupus vasculitis
Rheumatoid vasculitis
Sarcoid vasculitis
Vasculitis associated with probable etiology
Hepatitis C virus–associated cryoglobulinemic vasculitis
Hepatitis B virus–associated vasculitis
Drug‐associated immune complex vasculitis
Drug‐associated antineutrophil cytoplasmic antibody–associated vasculitis
Cancer‐associated vasculitis
Quoted from Reference (1), partially modified.

Small‐vessel vasculitis includes a total of seven diseases: microscopic polyangiitis, eosinophilic granulomatosis with polyangiitis, and granulomatosis with polyangiitis, which are antineutrophil cytoplasmic antibody (ANCA)–related vasculitides; IgA vasculitis; cryoglobulinemic vasculitis; hypocomplementemic urticarial vasculitis; and anti‐glomerular basement membrane disease, in which immune complexes are involved. Medium‐vessel vasculitis includes polyarteritis nodosa and Kawasaki disease, while large‐vessel vasculitis includes giant cell arteritis and Takayasu arteritis. Of these diseases, the three ANCA‐related vasculitides in small‐vessel vasculitis, IgA vasculitis, and cryoglobulinemic vasculitis are closely related to skin ulcers as their possible causes. Also notable are polyarteritis nodosa at the medium‐vessel level and cutaneous leukocytoclastic angiitis and cutaneous arteritis (corresponding to cutaneous polyarteritis nodosa) among single‐organ vasculitides.

In skin ulcers suspected to be due to vasculitis, histopathological findings by skin biopsy are extremely important for the definitive diagnosis. When performing a skin biopsy, the sampling sites should be carefully evaluated; if possible, samples should be obtained from several sites. However, skin ulcer sites are usually densely infiltrated by inflammatory cells including neutrophils; even if necrotizing vasculitis is present, its histological profile is difficult to detect in samples including tissue from the ulcerated area. Moreover, as a skin biopsy may enlarge or exacerbate skin ulcers, caution is necessary.

Meanwhile, livedo reticularis, nodules, infiltration, and tender erythema or purpura are often observed in addition to skin ulcers as skin symptoms of vasculitis. Therefore, in conducting a skin biopsy of the diagnosis of skin ulcers suspected to be due to vasculitis, histological evidence of necrotizing vasculitis is more likely to be obtained by carefully evaluating rashes other than ulcers. Also, if signs of necrotizing vasculitis cannot be detected in conventional skin biopsy specimens, they may be revealed in sections other than those examined. Therefore, it may also be necessary to examine deep‐cut sections in certain cases.

**FIGURE 4:**
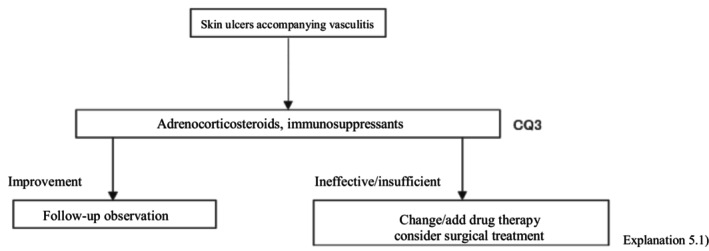
Therapeutic algorithm for vasculitis‐associated skin ulcers. CQ, clinical question.

The present guidelines mainly discuss skin ulcers in primary (idiopathic) vasculitis by excluding secondary vasculitis due to cancer, infections, or drugs. The therapeutic algorithm is shown in Figure 4, and the details are described in CQ3 and Explanation 5. In evaluating treatments for skin ulcers due to vasculitis, the manner of controlling the activity of the primary disease is important. While the present guidelines also mention treatments for vasculitis itself, see the “Guidelines for vasculitis and vasculopathy” by the Japanese Dermatological Association^2^ for details.

#### 2.2.5 RA‐associated skin ulcers

Causes of RA‐associated skin ulcers are broadly divided into “vasculitic” and “nonvasculitic.” The nonvasculitic skin ulcers can be further classified into “lower leg ulcers due to venous stasis,” “ulcers due to ischemic necrosis of soft tissue associated with compression,” “traumatic ulcers based on skin fragility,” and “other diseases that are likely to complicate rheumatoid arthritis.” Pyoderma gangrenosum is also likely to complicate RA and cause skin ulcers. Therefore, in treating skin ulcers in RA patients, it is important to differentiate among these causes and diseases. Regarding the differentiation procedure, it is important to first definitively differentiate between vasculitic and nonvasculitic ulcers because their treatments differ.

RA‐associated vasculitides is collectively called “rheumatoid vasculitis,” which is characterized by the marked diversity of the affected vascular levels compared with other vasculitides (polyarteritis nodosa, IgA vasculitis, granulomatosis polyangiitis, and eosinophilic granulomatosis polyangiitis; Table 2). Thus, rheumatoid vasculitis has characteristics of both necrotic vasculitis of the small arteries in the adipose tissue and leukocytoclastic vasculitis of the small veins in the dermis. (In Japan, RA accompanied by the former is customarily called “malignant rheumatoid arthritis” and the latter is called “rheumatoid vasculitis” in a narrow sense; internationally, all cases of RA‐associated vasculitides including both are called “rheumatoid vasculitis.”)

Therefore, its clinical symptoms are diverse, and all kinds of rash suggestive of vasculitis may occur, including subcutaneous nodules, livedo reticularis, skin ulcers, palpable purpura, hemorrhagic blisters, red papules, red nodules, scarlatiniform eruption, macular erythema, purpura, and white atrophy. If such skin symptoms are observed in RA patients, a skin biopsy is essential for the definitive diagnosis, as the accurate identification of the vascular level affected by vasculitis contributes to the selection of appropriate treatments and the determination of the prognosis.

Rheumatoid vasculitis may cause various extraarticular manifestations of RA, such as interstitial pneumonia, gastrointestinal lesions, cardiac lesions, and mononeuritis multiplex, in addition to the cutaneous symptoms. The frequency of the complication of RA by rheumatoid vasculitis is reportedly 0.7% to 5.4%.^3^, ^4^, ^5^, ^6^ Rheumatoid vasculitis is usually observed in a period in which RA activity is enhanced and in patients with a long history of illness (mean illness duration, 10–17 years) such as those with advanced joint destruction. This occurs more frequently in males than in females, as well as in those with high rheumatoid factor levels. Skin symptoms are observed in approximately 75% to 89% of patients with rheumatoid vasculitis,^7^, ^8^, ^9^, ^10^, ^11^, ^12^ and they often lead to its diagnosis because they are the initial extraarticular manifestations. Because the prognosis of rheumatoid vasculitis accompanied by systemic symptoms is poor, it is extremely important to invariably perform skin biopsy and ensure early diagnosis and early treatment if suspicious skin lesions are observed.

On the other hand, many of the causes of nonvasculitic skin ulcers are characterized by circulatory disturbances. Even in the absence of vasculitis, many blood vessels are known to degenerate regardless of their size or whether they are arteries or veins in the skin of RA patients, potentially leading to “lower leg ulcers due to venous stasis,” “ulcers due to ischemic necrosis of soft tissue associated with compression” (caused by joint deformity or contracture or inappropriate application of orthoses), and “traumatic ulcers based on skin fragility.”

**FIGURE 5:**
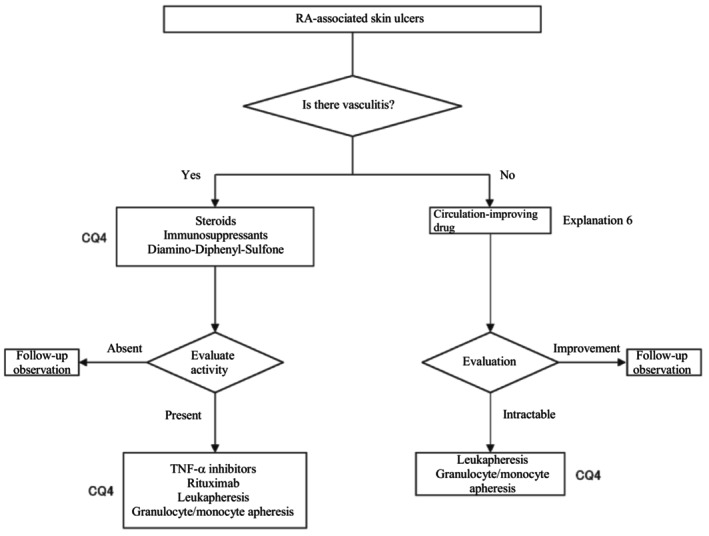
Therapeutic algorithm for rheumatoid arthritis (RA)–associated skin ulcers. CQ, clinical question; TNF‐α, tumor necrosis factor α.

In the present guidelines, a therapeutic algorithm (Figure 5) was prepared for the treatment for RA‐associated skin ulcers based on the above considerations, and the details are described in CQ4 and Explanation 6.

### 2.3 APS‐associated skin ulcers

APS is diagnosed when the patient tests positive for antiphospholipid antibodies and develops arterial or venous thrombosis or female infertility. Because arterial or venous thrombosis occurs in the skin and subcutaneous tissue, various skin symptoms develop and intractable skin ulcers frequently form.

Symptoms often observed in APS include arterial or venous thrombosis of the brain, heart, lungs, and limbs; recurrent miscarriage; thrombocytopenia; psychiatric or neurological symptoms, such epilepsy; skin symptoms; eye symptoms such as thrombosis of the central retinal artery or vein; and liver or kidney disorders. Among them, skin lesions are important as the initial manifestations of APS. Frances et al.^13^ evaluated 200 patients with APS and reported that skin lesions were observed in 31% of patients as the initial manifestations and in 49% throughout the disease course, and that livedo reticularis was the most frequently reported rash (observed in 25% of all patients). Similarly, Cervera et al.^14^ evaluated 1000 cases of APS and reported that skin lesions were observed as the initial manifestations in 29% and throughout the disease course in 40% of patients, and that livedo reticularis and necrosis due to skin infarction were noted in 24% and 5.5% of patients, respectively. Therefore, as skin lesions are the initial manifestations in many patients with APS, an aggressive examination for the presence or absence of APS should be performed if livedo reticularis or skin ulcers are present even without thrombosis of other organs.

Other cutaneous symptoms of APS reported to date include gangrene, subungual hemorrhage, pyoderma gangrenosum‐like rash, fulminant purpura, acrocyanosis, Raynaud phenomenon, Degos disease–like rash, and macular atrophy.^15^ In addition, reticular purpura observed in the lower leg is not specific to APS but should be differentiated from livedo vasculopathy, polyarteritis nodosa, cutaneous arteritis, cutaneous leukocytoclastic vasculitis, rheumatoid vasculitis, cryoglobulinemic vasculitis, hypergammaglobulinemic purpura, protein C/S deficiency, erythema induratum of Bazin, and syphilis. If livedo reticularis is observed in addition to intractable skin ulcers, the differentiation from the above diseases is difficult based on the clinical profile alone, and the cause must be evaluated by blood tests and histological examinations. While examinations that could risk enlarging the ulcerated surface are typically avoided if there are painful ulcers, a skin biopsy is essential to clarify whether thrombosis or vasculitis is involved in the cause of skin ulcers, and for evaluating the depth and thickness of the affected blood vessels. Because this examination is invasive, it is preferable to collect reliable tissue samples from the epidermis to the adipose tissue in as few attempts as possible, avoiding unnecessary repetition.

**FIGURE 6:**
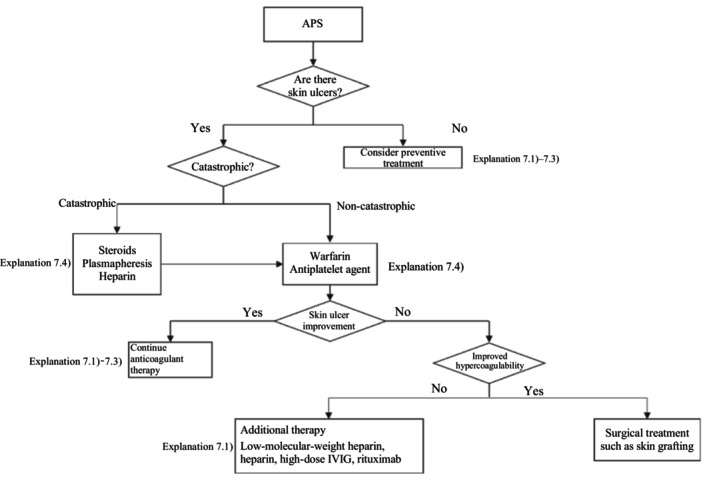
Therapeutic algorithm for antiphospholipid antibody syndrome (APS)–associated skin ulcers. IVIG, intravenous immunoglobulin.

A therapeutic algorithm based on the above principles is shown in Figure 6, and the details are described in Explanation 7. Anticoagulants are the mainstay treatment for APS. The concomitant use of circulation‐improving drugs is occasionally effective and worth attempting. Steroid administration may induce hypercoagulability but may also be useful for treating ulcers by suppressing secondary inflammation associated with skin ulcer formation. However, there is no consensus on the use of steroids, and the matter remains open to debate.

Because the control of APS itself is indispensable for treating associated skin ulcers, part of the Guidelines for vasculitis and vasculopathy by the Japanese Dermatological Association,^2^ MHLW Grants‐in‐Aid for Scientific Research (Intractable Disease Policy Research Project) for Research on Intractable Vasculitis Guidance for treatment of APS, eosinophilic granulomatosis with polyangiitis, polyarteritis nodosa, and rheumatoid vasculitis 2020^16^ was cited in the compilation of the present guidelines.

## 3 CHAPTER 3: GUIDELINES FOR THE MANAGEMENT OF CONNECTIVE TISSUE DISEASE/VASCULITIS‐ASSOCIATED SKIN ULCERS, CQS, AND RECOMMENDATIONS

**CQ1: Are there any drugs effective in preventing or treating SSc‐associated skin ulcers?**

**Table:** 

	Recommendation level	Description of recommendation	Results of voting
Prevention	Bosentan: Strong recommendation PDE5 inhibitors: weak recommendation	The prophylactic administration of bosentan for SSc‐associated skin ulcers is recommended. The prophylactic administration of PDE5 inhibitors (sildenafil, tadalafil) for SSc‐associated skin ulcers is proposed	Bosentan: strong recommendation 6/6 PDE5 inhibitors: weak recommendation 6/6
Treatment	Bosentan: weak recommendation PDE5 inhibitors: weak recommendation	Treatment with bosentan or PDE5 inhibitors (sildenafil, tadalafil) for SSc‐associated skin ulcers is proposed.	Bosentan: weak recommendation 6/6 PDE5 inhibitors: weak recommendation 6/6

### 3.1 Background/purpose

SSc induces fibrosis of the skin and various organs such as the lung/esophagus/intestinal tract/kidney. Concerning its pathogenesis, 3 factors are recognized: immune dysfunction, vascular damage, and fibrosis are primarily known, but vascular damage may play a key role in the onset of skin ulcers. Skin ulcers occur through persistent dermal ischemia related to microcirculatory disorder at the distal portions of the extremities such as the fingertips. Therefore, drugs that reduce vascular damage are selected as treatment options. Furthermore, in addition to skin ulcers, Raynaud phenomenon is observed as a representative symptom of vascular damage. This phenomenon refers to transient ischemia of the fingers related to reversible arterial spasm. Vasoconstrictors, such as endothelin, and cold stimulation–related sympatheticotonia, are complexly involved in the pathogenesis of this phenomenon. From the viewpoint of a similar pathogenesis, drugs that relieve Raynaud phenomenon are also regarded as candidates for treatment. Concretely, these include vasodilators, antiplatelet drugs, drugs for pulmonary hypertension, calcium antagonists, angiotensin‐converting enzyme (ACE) inhibitors, and statins. In the present guidelines, these treatment options were examined using the evidence‐based method (EBM) method. In addition, SSc‐associated skin ulcers are often refractory and can induce severe pain. Recurrent ulcers and shortening of the distal portions of the extremities may significantly affect the patient's QOL. From these viewpoints, the prevention of new skin ulcers is an important clinical issue, and drugs that prevent their onset were also investigated.

### 3.2 Scientific basis

To investigate drugs effective in preventing and treating skin ulcers in patients with SSc, randomized controlled trials (RCT) on “the prevention of new skin ulcers” and “healing of skin ulcers that had existed” were extracted, and 26 reports corresponded to them. Of these, bosentan and PDE5 inhibitors (sildenafil, tadalafil), of which quantitative systematic reviews had been performed, were adopted for this CQ.

Two RCTs^17,18^ showed that an endothelin receptor antagonist, bosentan, prevented the onset of new skin ulcers. In addition, a meta‐analysis^19^ reported its preventive effects on the onset of new skin ulcers. Thus, the recommendation level for prevention is strong. On the other hand, these RCTs did not show any significant difference in the curative effects on skin ulcers. There was no other drug with a high level of evidence on skin ulcer healing. We decided to propose treatment with bosentan through a review at a panel meeting, considering the publication of many case series studies of bosentan^20^, ^21^ and widespread clinical use of this drug for the treatment of refractory SSc‐associated skin ulcers in Japan. As there is no evidence exceeding the case series studies, the strength of total evidence is D (very weak), and the recommendation level is weak.

Concerning PDE5 inhibitors, there is one RCT of sildenafil^22^ showing a tendency to prevent the onset of new skin ulcers. Furthermore, one RCT of tadalafil^23^ showed that the number of patients with new ulcer onset was smaller than in the placebo group. Based on these results, the recommendation level for prevention is weak. With respect to therapeutic effects, one RCT of sildenafil demonstrated its therapeutic effects, and one RCT of tadalafil also showed its therapeutic effects on skin ulcers. The present guidelines drafting committee uniquely performed a meta‐analysis of these reports. Significant therapeutic effects on skin ulcers were observed. Considering a bias for these, the recommendation level for treatment is weak.

### 3.3 Commentary

Concerning the usefulness of the endothelin receptor antagonist bosentan, Korn et al.^17^ performed an RCT in 122 patients with SSc. In the bosentan group, the mean number of new ulcers was significantly inhibited in comparison with the placebo group. In addition, another RCT in 188 patients (Matucci‐Cerinic et al.^18^) showed a similar result. Tingey et al.^19^ performed a meta‐analysis of these RCTs and found the significant preventive effects of bosentan on new skin ulcers. In these RCTs, the therapeutic effects of bosentan on skin ulcers were not significant. On the other hand, Garcia et al.^20^ administered bosentan to 15 SSc patients with skin ulcers in a case series study, and confirmed a significant decrease in the number of skin ulcers, with a mean follow‐up of 24.7 months. Furthermore, a study reported the usefulness of this drug for treating skin ulcers other than digital ulcers.^21^ Concerning another endothelin receptor antagonist, macitentan, there is one RCT by Khanna et al.^24^ There was no significant difference in its preventive effects on the onset of new skin ulcers. However, in this trial, the incidence of new skin ulcers in the placebo group was lower than in other studies, and this may have contributed to the finding that there was no significant difference.

Concerning the PDE5 inhibitor sildenafil, Hachulla et al.^22^ performed an RCT in 83 patients with SSc‐associated skin ulcers (*n* = 192), and reported that the number of patients who developed new skin ulcers in the sildenafil group was slightly smaller than in the placebo group. Furthermore, sildenafil administration significantly decreased the mean number of skin ulcers. Shenoy et al.^23^ performed a crossover RCT of another PDE5 inhibitor, tadalafil, in 24 patients, and found that the results for the onset of new skin ulcers and skin ulcer healing were significantly better than in the placebo group. A meta‐analysis regarding the therapeutic effects of PDE5 inhibitors (sildenafil, tadalafil) on skin ulcers by Tingey et al.^19^ showed significant increases in the number of patients in whom the administration of PDE5 inhibitors reduced skin ulcers and the number of patients in whom healing was achieved. In the present guidelines, a meta‐analysis of a report from Hachulla et al.^22^ and four RCTs^25^, ^26^, ^27^ was uniquely performed with respect to skin ulcer healing. As a result, significant effects on skin ulcer healing as an outcome (relative risk [RR], 1.57 [95% CI, 1.14–2.17], *Z* = 2.76; *p* = 0.006) were observed in the PDE5 inhibitor–treated group. However, the number of meta‐analysis patients was approximately 60, and there may be a publication bias; thus, the strength of total evidence is B (middle), and the recommendation level is weak. Furthermore, a unique meta‐analysis regarding the preventive effects of PDE5 inhibitors on skin ulcers was performed based on reports from Hachulla et al.^22^ and Shenoy et al.,^23^ establishing the onset of new skin ulcers as an outcome. As a result, the onset of skin ulcers was significantly prevented in the PDE5 inhibitor–treated group (RR, 0.44 [95% CI, 0.22–0.89], *Z* = 2.30; *p* = 0.02). Considering the number of patients (approximately 60) and a publication bias, the strength of total evidence is B (middle), and the recommendation level is weak.

Regarding the following drugs, we concluded that RCTs did not confirm their preventive or curative effects on the onset of new skin ulcers or that the level of evidence from RCTs was low: beraprost sodium, lipo‐PGE1 preparations, nifedipine, atorvastatin, iloprost, udenafil, quinapril, cyclophosphamide, selexipag, treprostinil, botulinum toxin, and riociguat.

However, in clinical practice, prostaglandin preparations, calcium antagonists, and antiplatelet drugs have been empirically used for the treatment of SSc‐associated skin ulcers. In particular, many studies reported their usefulness for treating Raynaud phenomenon as a vascular lesion.^28^, ^29^, ^30^ Combination therapy with these drugs is also considered for the prevention and treatment of SSc‐associated skin ulcers in clinical practice (refer to Explanation 1, Chapter 5).

Furthermore, caution is needed for the use of ACE inhibitors or angiotensin II receptor antagonists. With respect to the effects of the former on vascular lesions, an RCT with quinapril showed that there was no improvement in the frequency or severity of Raynaud phenomenon, and that new skin ulcer onset was not inhibited.^31^ Concerning angiotensin II receptor antagonists, a comparative study of losartan potassium with nifedipine for Raynaud phenomenon was performed. There were slight improvements in the frequency and severity of Raynaud phenomenon in patients with SSc, but they were not significant. The two drugs did not significantly ameliorate Raynaud phenomenon as a vascular lesion.^32^ In addition, ACE inhibitors are effective for scleroderma renal crisis, but their usefulness in prophylactic administration remains to be clarified.^31^, ^33^ Furthermore, a recent large‐scale, prospective cohort study in Europe showed that the administration of ACE inhibitors rather increased the risk of scleroderma renal crisis.^34^ Based on these findings, if other drugs are available, the introduction of ACE inhibitors only for the prevention and treatment of skin ulcers should be carefully examined.

### 3.4 Precautions for clinical use

Among the recommended drugs, bosentan administration for preventing the onset of new skin ulcers is covered by health insurance. As an adverse reaction, the incidence of liver dysfunction is high, and there are serious cases; therefore, caution is required. On the other hand, bosentan administration for treating skin ulcers is not covered by health insurance. PDE5 inhibitors are covered by health insurance only for pulmonary arterial hypertension. Therefore, whether the recommended drugs are indicated must be carefully considered.

### 3.5 Possibility of future research

There are many articles describing the usefulness of calcium antagonists for treating SSc‐related Raynaud phenomenon^30^, ^35^ These agents may reduce peripheral circulatory disorder. On the other hand, there is only one RCT on the prevention of new skin ulcer onset and healing.^36^ No RCT of which the evidence level is high has been performed. Calcium antagonists may be useful for preventing the onset of new skin ulcers and achieving skin ulcer healing in patients with SSc, but may this has not been sufficiently evaluated. As they are frequently used in clinical practice, evidence should be established in the future. Concerning botulinum, there is one RCT each on botulinum toxin type A/B with respect to the prevention of new skin ulcer onset and healing.^37^, ^38^ Botulinum toxin type B was confirmed to inhibit the onset of new skin ulcers and promote skin ulcer healing. Currently, it is not covered by health insurance, but its effects should be demonstrated by accumulating cases in the future as a remedy from Japan.

**CQ2: Are there any treatment methods effective for connective tissue disease–associated calcinosis cutis?**

**Table:** 

Recommendation level	Description of recommendation	Results of voting
Weak recommendation for all treatments	Treatment with warfarin, diltiazem hydrochloride, colchicine, or bisphosphonate preparations or by surgical resection for connective tissue disease–related calcinosis cutis is proposed. Treatment with rituximab for SSc‐related calcinosis cutis is proposed. It is proposed to avoid treatment with rituximab for dermatomyositis‐related calcinosis cutis	Warfarin: Connective tissue disease, weak recommendation 7/7 Diltiazem hydrochloride: connective tissue disease, weak recommendation 7/7 Colchicine: connective tissue disease, weak recommendation 7/7 Bisphosphonate preparations: connective tissue disease, weak recommendation 7/7 Surgical resection: connective tissue disease, weak recommendation 7/7 Rituximab: SSc, weak recommendation 7/7 Dermatomyositis: weak recommendation 7/7

### 3.6 Background/purpose

In patients with connective tissue disease, dermal to subcutaneous calcinosis is often observed. Among various types of connective tissue diseases, calcinosis is most commonly induced by SSc (18%–49%) and dermatomyositis (in adults: ≤30%, in children: 30%–70%).^39^, ^40^, ^41^, ^42^, ^43^, ^44^, ^45^, ^46^, ^47^, ^48^ Calcinosis cutis often causes ulceration through bacterial infection–related secondary infection or self‐destruction. Furthermore, calcinosis may induce pain, leading to a limitation in the range of motion and muscle atrophy. For skin ulcers that follow calcinosis, general drugs for skin ulcers alone are not effective, and calcification‐targeting treatment is necessary. In clinical practice, various drugs, including warfarin and diltiazem hydrochloride, are used to treat small, calcified lesions. Furthermore, surgical resection is sometimes considered for large, extensive calcified lesions, considering invasiveness. Therefore, the treatment of calcinosis cutis with pain or ulceration is clinically an important issue, and we aim to examine the effects of each treatment method on connective tissue disease–related calcification using the EBM method and present the directivity of treatment.

### 3.7 Scientific basis

To verify drugs effective in reducing and inhibiting connective tissue disease–related calcinosis, a literature search on various treatment methods was performed. With respect to treatment methods (warfarin, diltiazem hydrochloride, colchicine, bisphosphonate preparations, surgical resection, rituximab) with evidence above case series studies for SSc and dermatomyositis among various types of connective tissue disease, the literature was adopted, and the recommendation level was described.

The literature review consisted of: warfarin (one RCT,^49^ two case series studies),^50^, ^51^ diltiazem hydrochloride (two cohort studies,^52^, ^53^ two case series studies),^54^, ^55^ colchicine (two cohort studies),^52^, ^53^ bisphosphonate preparations (one cohort study),^55^ surgical resection (two cohort studies,^52^, ^53^ one case series study),^56^ rituximab (SSc, three case series studies),^57^, ^58^, ^59^ and dermatomyositis (one RCT,^60^ one cohort study,^52^ one case series study).^61^

### 3.8 Commentary

Warfarin inhibits a vitamin K–dependent enzyme that converts glutamic acid to γ‐carboxyglutamic acid in the process of calcification; it may have anticalcification actions.^62^ Concerning the effects of warfarin on calcinosis cutis, there is one RCT^49^ and two case series studies.^50^, ^51^ Berger et al.^49^ performed a pilot study in four patients with connective tissue disease and calcinosis cutis, including two with dermatomyositis and one with overlap syndrome consisting of dermatomyositis/SSc, to examine the effects of low‐dose (1 mg/day) warfarin administration for 18 months. As a result, there was a decrease in the urinary γ‐carboxyglutamic acid level in two patients, and systemic scintigraphy showed a decrease in subcutaneous uptake of Tc‐99m diphosphate. In one patient, there was a decrease in the number of calcified lesions. Subsequently, four patients were newly added, and the effects of low‐dose (1 mg/day) warfarin were investigated for 18 months in a total of eight patients. One patient dropped out of the study because of poor compliance, and the study was continued in the other seven patients. Although there were no changes in calcified lesions, there was a decrease in Tc‐99m diphosphate uptake on systemic scintigraphy in two‐thirds of the patients in the warfarin‐treated group. In the placebo group, there was no decrease in Tc‐99m diphosphate uptake. As there was no influence on the bleeding time or prothrombin time, it was concluded that warfarin was useful for inhibiting the progression of calcification. Furthermore, Cukierman et al.^50^ administered low‐dose (1 mg/day) warfarin to three patients with SSc for 1 year to treat calcified lesions and found that calcified lesions measuring ≤2 cm were ameliorated, with no adverse reactions such as bleeding tendency. On the other hand, Lassoued et al.^51^ administered 1 mg/day of warfarin to six patients with prolonged calcinosis (five with dermatomyositis and one with SSc) for 1 year and reported its ineffectiveness. For the following reasons, the strength of total evidence is D (very weak) and the recommendation level is weak. The two studies were observational and the RCT had a bias risk (−1).

Diltiazem hydrochloride may prevent calcinosis by inhibiting intracellular calcium ion influx. There are four case reports showing its effectiveness in treating calcification in patients with dermatomyositis^63^, ^64^, ^65^ or SSc.^66^ In addition, there are two cohort studies^52^, ^53^ and two case series studies^54^, ^55^ that provide further evidence. Fredi et al.^52^ performed a retrospective cohort study in 74 patients (30 with polymyositis, 30 with dermatomyositis, 13 with overlap syndrome, one with sporadic inclusion body myositis), and reported that diltiazem hydrochloride had been administered to seven of 16 patients with calcification, whereas there was no treatment response. Balin et al.^53^ performed a retrospective cohort study in 78 connective tissue disease patients with calcification. Of 14 diltiazem hydrochloride–treated patients in whom it was possible to evaluate the treatment response, there was no response in five and a partial response was achieved in nine. Vayssarirat et al.^46^ performed a case series study, and investigated the effects of diltiazem hydrochloride at 180 mg/day on SSc‐related calcified lesions. In three of 12 patients with SSc in whom image‐based assessment was possible, a slight improvement was achieved. Palmieri et al.^54^ administered diltiazem hydrochloride at 240 to 480 mg/day to four patients with idiopathic calcification and one with CREST syndrome, and reported improvements in all patients. As these were observational studies, the strength of total evidence is D (very weak), and the recommendation level is weak.

Colchicine may exhibit anticalcification actions by inhibiting leukocyte migration. Concerning the effects of colchicine on calcinosis cutis, there are two cohort studies.^52^, ^53^ Fredi et al.^52^ performed a retrospective cohort study and reported its effectiveness in one of nine patients. In another retrospective cohort study by Balin et al.,^53^ remission was achieved in one of seven patients, there was no response in four, and a partial response was achieved in two. As these were observational studies, the strength of total evidence is D (very weak), and the recommendation level is weak.

Concerning the effects of bisphosphonate preparations on calcinosis cutis, there is one cohort study.^55^ According to the study, treatment with bisphosphonate preparations in eight adult dermatomyositis patients with calcification resulted in remission in two, whereas there was no response in six. Thus, the strength of total evidence is D (very weak), and the recommendation level is weak.

Regarding the effects of surgical resection on calcinosis cutis, there are two retrospective cohort studies^52^, ^53^ and one case series study.^56^ Fredi et al.^52^ performed a retrospective cohort study in 74 patients (30 with polymyositis, 30 with dermatomyositis, 13 with overlap syndrome, one with sporadic inclusion body myositis). Of six patients who underwent surgical resection, it was effective in three. Balin et al.^53^ performed a retrospective cohort study in 78 connective tissue disease patients with calcified lesions (30 with dermatomyositis, 24 with SSc, six with overlap syndrome, four with mixed connective tissue disease, four with lupus panniculitis, two with SLE, one with RA, one with polymyositis, six with undifferentiated connective tissue disease). In 28 patients, surgery was performed, leading to “remission” in 22, “partial improvement” in five, and “no change” in one. They reported that surgery was useful for treating large, extensive, symptomatic calcified lesions. Bogoch et al.^56^ reviewed 34 articles in a case series study on surgery for the hands of patients with SSc, and reported that surgical resection reduced moderate pain, improving function. However, they reported the necessity of extended resection, a poor peripheral circulation–related delay in wound healing, necrosis, and possibility of a necrosis‐related limitation in the range of motion. Thus, the strength of total evidence is D (very weak), and the recommendation level is weak.

Concerning the effects of rituximab on calcinosis cutis, there are three case series studies in patients with SSc.^57^, ^58^, ^59^ Moazedi‐Fuerst et al.^57^ reported the disappearance of calcification in all three patients. Narvaez et al.^58^ observed “complete remission” in two of eight patients, “partial response” in two, and “no response” in four. Giuggioli et al.^59^ reported that three of six patients responded. As these were case series studies, the strength of total evidence is D (very weak), and the recommendation level is weak.

On the other hand, concerning dermatomyositis, there is one RCT,^60^ one cohort study,^52^ and one case series study.^61^ Aggarwal et al.^60^ administered rituximab to 120 patients with dermatomyositis (72 adults [seven with calcinosis], 48 young patients [22 with calcinosis]), and reported its effects on dermal lesions. As an item for evaluating the effects, they adopted calcinosis, but suggested that rituximab was ineffective. Bader‐Meunier et al.^61^ administered rituximab to nine patients with juvenile dermatomyositis (six with calcinosis), and reported that it was ineffective for calcinosis, whereas it was effective for dermal/muscular lesions to some extent. Fredi et al.^52^ administered this drug to two patients with dermatomyositis, and reported that one patient responded. As the RCT by Aggarwal et al. has a bias risk (−1), the strength of total evidence is D (very weak), and the recommendation level is weak.

### 3.9 Precautions for clinical use

In the literature adopted in this CQ, there are many cases in which combination therapy for connective tissue disease was selected or in which various treatments had been performed. For this reason, it was difficult to verify the efficacy of individual drugs. Furthermore, many clinical studies that we adopted examined the efficacy for organ lesions other than calcinosis lesions; therefore, the number of patients was small to investigate the efficacy for calcified lesions, and statistical analysis was impossible. Briefly, the evidence level of all treatments is low, and attention must be paid to adverse reactions to the respective drugs when selecting treatment. In clinical practice, recommended drugs, such as warfarin and diltiazem hydrochloride, should be sequentially selected for small calcified lesions. Surgical resection should be considered for large, extensive calcified lesions, considering invasiveness. However, surgical treatment is associated with some risks, such as a delay in wound healing, secondary infection, and pain, and should be performed in patients in whom the advantage of treatment may exceed its disadvantage. Furthermore, it is necessary to consider that the condition may often recur even if these treatments are performed.

In this CQ, the recommendation level is not described, but case series or cohort studies suggested the efficacy of some drugs for dermatomyositis‐ or SSc‐related calcinosis lesions. Concerning SSc, there is a case series study demonstrating the efficacy of minocycline. Robertson et al.^67^ administered 50 to 100 mg/day of minocycline to nine limited cutaneous SSc patients with calcified lesions, and reported that a partial response was achieved in eight patients. In dermatomyositis, anti–tumor necrosis factor α (TNF‐α) preparations,^68^ intravenous immunoglobulin therapy,^52^, ^69^ and intravenous cyclophosphamide therapy^70^ have been reported. Campanilho‐Marques et al.^68^ performed a cohort study regarding the efficacy of anti–TNF‐α preparations in 60 patients with childhood dermatomyositis, and reported their efficacy in 15 (54%) of 28 patients with calcified lesions and disappearance in eight (29%). For intravenous immunoglobulin therapy, there are two retrospective cohort studies.^52^, ^69^ Galimberti et al.^69^ found a partial response in five of eight patients with dermatomyositis. Fredi et al.^52^ reported that an improvement was achieved in one of seven patients. Overall, the efficacy of this therapy was demonstrated in six of 15 patients with dermatomyositis; it may be effective, although the evidence level is not high. Moraitis et al.^70^ performed a cohort study of intravenous cyclophosphamide therapy for severe childhood dermatomyositis, and reported that calcified lesions were reduced in nine of 14 patients.

### 3.10 Possibility of future research

Currently, there are no high‐quality RCTs establishing calcinosis as a primary outcome. There are only two studies (one RCT of warfarin in a small number of patients^49^ and one RCT of rituximab) establishing calcinosis as a secondary endpoint.^60^ In other case series or cohort studies, there is no sufficient description on the size of calcinosis lesions, severity, period, or pain, and subgroup analysis was not performed. In patients with connective tissue disease, calcinosis treatment is an important unmet needs. In the future, a high‐quality intervention study should be performed.

**CQ3: Are there any drugs effective for vasculitis‐associated skin ulcers?**

**Table:** 

Recommendation level	Description of recommendation	Results of voting
Weak recommendation	Systemic steroid administration is proposed because steroids are routinely used as a base drug for vasculitis‐associated skin ulcers in clinical practice, and because their effects have been obtained	Systemic steroid administration: Weak recommendation 7/7

### 3.11 Background/purpose

Vasculitis refers to a disease in which the vascular structure is damaged through immune cell infiltration in the vascular wall, inducing ischemia‐related tissue/organ disorder. The pathogenesis of vasculitis remains to be clarified. Diagnosis/treatment methods have not been sufficiently established. In the Chapel Hill classification^71^ published in 1994, the classification of vasculitis based on vascular thickness (large, medium, and small blood vessels) was proposed. In a revision in 2012,^1^ it was subdivided in consideration of the etiology and underlying disease. In addition, the Nomenclature of Cutaneous Vasculitis: Dermatological Addendum to the CHCC 2012^72^ was published in 2018, providing a detailed description of individual skin lesions associated with vasculitis. In the skin, the incidence of medium/small vessel vasculitis is high. Cutaneous vasculitis induces various dermal symptoms, but skin ulcers may occur.

For the treatment of vasculitis‐associated skin ulcers, it is the most important to control primary disease activity. However, there are cases in which it is difficult to evaluate which treatment is useful for treating skin ulcers, especially when physicians other than dermatologists are responsible for treatment. In this section, we establish the CQ “Are there any drugs effective for vasculitis‐associated skin ulcers?” and aim to present the directivity of treatment for vasculitis‐related skin ulcers based on the literature previously reported.

### 3.12 Scientific basis

Systemic steroid administration is inexpensive, effective, and fast‐acting; therefore, it is ranked as a first‐choice drug for the treatment of vasculitis‐associated skin ulcers,^73^ and routinely used. According to a study presented by Daoud et al.,^74^ in which 39 patients with cutaneous arteritis (conventionally termed “cutaneous polyarteritis nodosa”) with skin ulcers and 40 without skin ulcers were retrospectively investigated, the duration of disease was slightly prolonged in the former, and the incidence of concomitant neuropathy was high. Furthermore, prednisolone administration at 60 to 80 mg/day reduced pain, subcutaneous nodes, and skin ulcers in most patients with skin ulcers, but dose reduction led to recurrence in most patients. Combination therapy with immunosuppressants was attempted. Kumar et al.^75^ reported eight patients with childhood cutaneous arteritis and terminal gangrenes. Systemic steroid administration resulted in remission in all patients, but recurrence was noted in four patients and spontaneous falling of the fingers or toes in six, leading to amputation of the right leg in one patient. In addition, there are many case reports on polyarteritis nodosa,^76^ eosinophilic granulomatosis with polyangiitis,^77^ and granulomatosis with polyangiitis^78^ in which a combination of systemic steroid administration and immunosuppressants reduced skin ulcers.

### 3.13 Commentary

Although high‐level evidence in the literature is limited, as noted above, systemic steroid administration is routinely selected as a base remedy for vasculitis‐associated skin ulcers in clinical practice, and its effects have been reported. We considered it useful, but the recommendation level is weak.

Other drugs include cyclophophamide, rituximab, methotrexate, azathioprine, and intravenous immunoglobulin. Cyclophophamide is routinely used for remission induction and maintenance therapies in patients with polyarteritis nodosa, ANCA‐associated vasculitis, or giant cell arteritis. Many studies have reported on the therapeutic effects of combination therapy with systemic steroid administration for vasculitis‐associated skin ulcers.^78^, ^79^, ^80^, ^81^, ^82^, ^83^, ^84^, ^85^, ^86^, ^87^, ^88^ Rituximab was shown to be as effective as cyclophosphamide in performing remission induction therapy for ANCA‐associated vasculitis.^89^ Its usefulness for treating skin ulcers related to ANCA‐associated vasculitis has been reported.^90^, ^91^, ^92^ In an RCT in 57 patients with cryoglobulinemic vasculitis,^93^ rituximab administration led to the remission of skin ulcers in all five patients with skin ulcers. Concerning methotrexate, there are case reports showing its efficacy for steroid‐resistant polyarteritis nodosa–associated skin ulcers.^94^, ^95^ Several studies reported the efficacy of azathioprine in combination with systemic steroid administration for cutaneous arteritis– or polyarteritis nodosa–associated skin ulcers.^76^, ^96^ Concerning intravenous immunoglobulin therapy, there are case reports demonstrating its effectiveness for skin ulcers associated with refractory polyarteritis nodosa or granulomatosis with polyangiitis.^79^, ^97^, ^98^ Thus, systemic steroid administration or immunosuppressive therapy for vasculitis control may also be useful for treating vasculitis‐associated skin ulcers. However, with respect to individual drugs, there are few articles of which the evidence level is high, and they should be further examined in the future.

### 3.14 Precautions for clinical use

As long‐term systemic steroid administration induces various adverse reactions, such as infection, diabetes, hypertension, and osteoporosis, caution is needed. If sufficient treatment with steroids or immunosuppressants does not reduce skin ulcers, skin ulcer–deteriorating factors, such as thrombus formation, venous return disturbance, and concomitant infection, must also be considered. For the use of azathioprine, an NUDT15 gene polymorphism test must be performed to evaluate whether this drug should be indicated. Methotrexate is not covered by health insurance for vasculitis in Japan. For its use, caution is recommended.

### 3.15 Possibility of future research

Vasculitis is rare, and it may be difficult to accumulate a sufficient number of patients at a single institution. The literature primarily consisted of descriptive studies. In the future, the efficacy of each drug should be examined in a multicenter cooperative study, establishing skin ulcers as a primary endpoint. Furthermore, the condition may differ among types of vasculitis, and which immunosuppressant is effective for skin ulcers associated with respective disease types must be investigated in the future. With recent marked advances in vasculitis treatment, the efficacy of biological preparations, such as anti–interleukin 6 receptor antibody, Janus kinase (JAK) inhibitors, and C5a receptor inhibitors has been clarified. In the future, the efficacy of these new drugs for vasculitis‐associated skin ulcers should also be further examined.

**CQ4: Are there any treatment methods effective for rheumatoid vasculitis–associated skin ulcers?**

**Table:** 

Recommendation level	Description of recommendation	Results of voting
Weak recommendation for all drugs	Treatment with azathioprine in combination with steroids or alone, cyclophosphamide + steroid pulse, TNF‐α inhibitors, rituximab, or LCAP/GCAP for rheumatoid vasculitis–related skin ulcers is proposed	Azathioprine: weak recommendation 7/7 Cyclophosphamide + steroid pulse: weak recommendation 7/7 TNF‐α inhibitors: weak recommendation 7/7 Rituximab: weak recommendation 7/7 LCAP/GCAP: weak recommendation 7/7

### 3.16 Background/purpose

Rheumatoid vasculitis is a general term for RA‐related vasculitis. Clinically, heterogeneity is strong, and various blood vessels (small to large) are damaged. Vasculitis at various levels can be observed in the skin, ranging from leukocytoclastic vasculitis of dermal venules to necrotizing vasculitis of adipose tissue arterioles, often inducing skin ulcers. For the treatment of rheumatoid vasculitis–associated skin ulcers, standard drugs for skin ulcers are ineffective, and vasculitis‐targeting treatment is necessary. However, many treatment options, such as steroids, immunosuppressants, diaminodiphenyl sulfone (DDS), TNF‐α inhibitors, rituximab, leukocytapheresis/granulocytapheresis (LCAP/GCAP), and JAK inhibitors, are listed as candidates. It is often difficult to determine which treatment method should be used in clinical practice. We aim to present the directivity of treatment by examining the effects of each treatment method on rheumatoid vasculitis–associated skin ulcers using the EBM method.

### 3.17 Scientific basis

A literature search on drugs that are used for the treatment of rheumatoid vasculitis in Japan (steroids, cyclophosphamide + steroid pulse, azathioprine, DDS, TNF‐α inhibitors, rituximab, LCAP/GCAP, JAK inhibitors, methotrexate) was performed, and 15 articles verifying their usefulness for treating rheumatoid vasculitis–associated skin ulcers were adopted: two RCTs,^99^, ^100^ one non‐RCT,^101^ six cohort studies,^102^, ^103^, ^104^, ^105^, ^106^, ^107^ and six case series studies.^108^, ^109^, ^110^, ^111^, ^112^, ^113^ Statistical examination was not performed in any study.

### 3.18 Commentary

In the two RCTs identified in the literature search,^99^, ^100^ the efficacy of azathioprine for treating rheumatoid vasculitis was verified. In one, combination therapy with steroids was performed. In the other, azathioprine was combined with anti‐inflammatory analgesic drugs including steroids. The two studies have a high risk of bias (randomized –2, blind –2, intention‐to‐treat –2), and patients with skin ulcer‐free vasculitis were included, making it impossible to assess the impact on skin ulcers. Thus, the strength of total evidence is D (very weak). Furthermore, these RCTs did not show any significant effects of azathioprine on skin ulcers, recrudescence, or complications. However, in clinical practice, azathioprine is routinely used as a base drug for rheumatoid vasculitis, and there are many reports of its utility. Thus, the recommendation level is weak.

Concerning the effects of cyclophosphamide on rheumatoid vasculitis, there is only one case series study verifying the efficacy of oral administration.^108^ However, for combination therapy with cyclophosphamide and steroid pulse, there is one nonrandomized open study.^101^ Of 45 patients with rheumatoid vasculitis, cyclophosphamide + steroid pulse therapy was selected in 21 severe‐status patients, and conventional drugs (azathioprine, prednisolone, D‐penicillamine, chlorambucil, cyclophosphamide alone, methotrexate, prostaglandin) were used in the other 24 patients. Cyclophosphamide + steroid pulse therapy reduced skin ulcers in 67% of the patients, whereas an improvement was achieved in only 25% of the patients in the other treatment group. Furthermore, the incidence of recurrent skin ulcers was 10% in the cyclophosphamide + steroid pulse therapy group, whereas it was 33% in the other treatment group, suggesting the inhibitory effects of cyclophosphamide + steroid pulse therapy on recurrent skin ulcers. However, this study has a high risk of bias (randomized −2, blind −2, intention‐to‐treat −2, others −2). Thus, the strength of total evidence is D (very weak), and the recommendation level is weak.

For TNF‐α inhibitors, there was one cohort study^102^ and two case series studies^109^, ^110^ included. In the cohort study performed by Josselin et al.,^102^ infliximab was administered to three rheumatoid vasculitis patients with skin ulcers. Remission (Birmingham Vasculitis Activity Score: 0) was achieved in two patients, and a treatment response (rate of decrease in the Birmingham Vasculitis Activity Score: ≥50%) was achieved in one patient. Puechal et al.^109^ performed a case series study, and reported that “complete remission,” “partial remission,” and “no response” were achieved in three, one, and one of five infliximab‐ or etanercept‐treated rheumatoid vasculitis patients with skin ulcers, respectively. In the case series study performed by Bartolucci et al.,^110^ only one patient had rheumatoid vasculitis with skin ulcers. In this patient, the administration of a TNF‐α inhibitor led to partial remission after 42 days and ulcer healing after 6 months. As these were observational studies, the strength of total evidence is D (very weak), and the recommendation level is weak.

Regarding rituximab, there were two cohort studies^103^, ^104^ and one case series study^111^ included. According to a report from Coffey et al.,^103^ “complete remission,” “partial remission,” and “no response” were achieved at 6 months in three, three, and one of seven rheumatoid vasculitis patients with skin ulcers who were assessed at follow‐up, respectively. After 12 months, complete remission was achieved in five and partial remission in two, showing good efficacy. Furthermore, Puechal et al.^104^ also reported that the complete remission rate 12 months after the start of rituximab administration was 82%, suggesting its efficacy. However, in these studies, there were patients with concomitant infection, including those who died. Thus, as there is no RCT of rituximab, the strength of total evidence is D (very weak), and the recommendation level is weak.

Concerning LCAP/GCAP, there were three cohort studies^105^, ^106^, ^107^ and one case series study.^112^ All studies showed an improvement in skin ulcers, and there was no serious adverse reaction or complication. However, patients in whom the etiology of ulcers was unclear were included.^105^ The bias risk of each study is high, as represented by combination therapy with immunosuppressants.^107^ Furthermore, in these reports, the number of rheumatoid vasculitis patients with skin ulcers was small (Mori et al.: three patients, Hidaka et al.: two patients, Winkelstein et al.: six patients, Itoh et al.: three patients). Thus, the strength of total evidence is D (very weak), and the recommendation level is weak.

Concerning steroids, JAK inhibitors, and methotrexate, their efficacy for rheumatoid vasculitis–associated skin ulcers and safety were verified only in case reports. Concerning DDS, there is one case series study,^113^ but it involved only two patients. Neither RCTs nor cohort studies of these drugs were adopted.

### 3.19 Precautions for clinical use

In many patients with rheumatoid vasculitis–associated skin ulcers included in this CQ, some form of medication had already been administered at the start of ulcer treatment under a diagnosis of RA. In most of the clinical studies reviewed at this time, several drugs, including steroids, had also been administered. This made it extremely difficult to verify the efficacy of individual drugs. Furthermore, many of the above clinical studies examined the efficacy of treatments for rheumatoid vasculitis, and patients with skin ulcers were limited; the number of patients was small, and statistical analysis was not possible.

Concerning steroids, there is no clinical study to evaluate their efficacy for the treatment of ulcers, and this could not be verified in this CQ. However, steroids have been routinely used as a first‐choice drug for rheumatoid vasculitis for many years. There are case series studies demonstrating the efficacy of steroids^7^, ^114^; therefore, their effects are indisputable. In the present guidelines, the recommendation level is not presented, but systemic steroid administration should also be considered as an option for rheumatoid vasculitis–associated skin ulcers.

In clinical practice, physicians are responsible for determining treatment, taking into account both the efficacy for rheumatoid vasculitis, as an underlying cause of the ulcers, and the treatment of the skin ulcers themselves. DDS, methotrexate, and JAK inhibitors, which may be effective for rheumatoid vasculitis, in addition to steroids, may also be available as treatment options.

### 3.20 Possibility of future research

With respect to treatment for rheumatoid vasculitis, there are many options, but no intervention study has investigated the efficacy of a drug/treatment method for ulcers as a primary outcome. Currently, there are only observational studies, of which the evidence level is low. A high‐quality RCT should be performed. Recently, JAK inhibitors became commercially available as a new drug for RA or vasculitis. The efficacy of JAK inhibitors was verified, establishing articular symptoms of RA as an outcome. However, no clinical study regarding rheumatoid vasculitis with skin ulcers was extracted. In Japan, the frequency of use has increased since approval in 2013. Therefore, if the efficacy for rheumatoid vasculitis–associated skin ulcers is examined, and if a comparative study with conventional treatment methods is performed, a clear answer to the question “which drug should be recommended for rheumatoid vasculitis–associated skin ulcers?” may be obtained.

## 4 CHAPTER 4: DEFINITIONS OF TERMINOLOGY

The terminology used in these guidelines was defined as indicated below based on the contents of review papers and textbooks in Japan. In addition, these guidelines partially quote the terminology list of the Terminology Committee of the Japanese Society of Pressure Ulcers, and consideration was made for consistency within the guidelines.

### 4.1 Topical agents

Drugs that are applied through the skin or directly to skin lesions for localized treatment. They are prepared by compounding various drugs with a base.

### 4.2 Dressing materials

Modern wound‐dressing materials for creating a moist environment for wounds. Conventional sterile gauze is excluded.

### 4.3 Wound‐dressing materials

Wound‐dressing materials can be broadly classified into dressing materials (modern dressing materials) and medical materials such as gauze (classic dressing materials). The former are medical materials that provide conditions optimal for wound healing by maintaining a moist environment and must be used selectively depending on the state of the wound and amount of exudates. The latter allow drying of the wound and cannot maintain a moist environment if exudates are insufficient. Medical materials other than conventional gauze that provide an optimal environment for wound healing by covering the wound and maintaining moisture may also be called wound‐dressing materials or dressing materials.

### 4.4 Surgical treatments

Surgical therapy, surgical debridement, and invasive procedures of subcutaneous pockets.

### 4.5 Debridement

A therapeutic action used to clean a wound by removing foreign material, necrotic tissue, and senescent cells that no longer react to stimulation by promoters of wound healing such as growth factors and foci of bacterial infections, which are often associated with the above. Methods include: (i) autolytic debridement induced by occlusive dressing; (ii) mechanical debridement (e.g. wet‐to‐dry dressing, high‐pressure washing, hydrotherapy, and ultrasonic washing); (iii) debridement using proteolytic enzymes; (iv) surgical debridement; and (v) biological debridement using maggots.

### 4.6 Wet‐to‐dry dressing

Dressing aimed at debridement performed by applying gauze saturated with physiological saline to the wound, and, once the gauze has dried, nonselective removing of foreign material and necrotic tissue adhering to it occurs when it is changed.

### 4.7 Occlusive dressing

All dressing methods used to avoid wound drying in moist wound healing are called occlusive dressings. This is a collective term for dressing using modern wound‐dressing materials other than conventional gauze dressings.

### 4.8 Wound bed preparation

Management of the wound surface environment to promote wound healing. Specifically, necrotic tissue is removed, the bacterial load is reduced, wound drying is prevented, excessive exudates are controlled, and pockets and wound margins are treated.

### 4.9 Moist wound healing

Maintaining a moist wound surface. This retains multinucleated leukocytes, macrophages, enzymes, and cell growth factors within exudates on the wound surface. Such an environment promotes autolysis and the removal of necrotic tissue and does not interfere with cell migration.

### 4.10 Raynaud phenomenon

Finger/toe vasospasm triggered by cold or psychological stress, causing clearly bordered color changes in the fingers/toes. Three‐phase color changes of white–purple–red are typically observed.

### 4.11 Finger/toe tip ulcer

Skin ulcer occurring at the end of a finger or toe primarily due to peripheral circulatory disorders. This usually painful condition occurs frequently in patients with SSc but is also observed even in patients who show no skin sclerosis and do not satisfy the diagnostic criteria for SSc.

### 4.12 Calcinosis of the skin

Calcinosis is often observed from the dermis to subcutis in patients with connective tissue diseases including SLE, SSc, and dermatomyositis. Calcinosis of the skin is generally classified as: (i) “metastatic” calcification; (ii) tumoral calcification; (iii) dystrophic calcification; (iv) idiopathic calcification; and (v) calciphylaxis. (i) is accompanied by abnormal blood calcium and phosphorus concentrations and is due to hyperparathyroidism, malignant tumor, so‐called “milk‐alkali syndrome,” or excessive vitamin D intake. (ii) is a rare familial disease exhibiting elevated blood phosphorus and normal calcium concentrations and causing very large calcium deposits in the joints and at sites of compression. (iii) is calcinosis observed at sites of injury showing no particular abnormalities in blood calcium or phosphorus concentrations. It occurs frequently after trauma or infection and in patients with connective tissue diseases such as SLE, SSc, and dermatomyositis. This may affect various sites including the limbs and buttocks. (iv) is single or multiple subcutaneous calcium deposits observed in healthy individuals without metabolic abnormalities. (v) is calcinosis of the vascular wall accompanied by abnormal blood calcium/phosphorus concentrations and secondary ischemia/necrosis of the skin observed in patients with chronic renal insufficiency. Calcinosis in patients with connective tissue diseases is related to a nutritional disturbance preceded by a systemic underlying disorder corresponding to (iii) dystrophic calcification. While the mechanism of calcium deposition in tissues remains largely unclear, local inflammation and circulatory disorders are possible causes.

### 4.13 Lupus erythematosus profundus (lupus panniculitis)

Lupus erythematosus (LE) profundus is a variation of LE with lesions seated primarily in the adipose tissue. It is synonymous with LE panniculitis, but caution is necessary because panniculitis alone is occasionally distinguished as LE panniculitis from LE profundus, a state in which panniculitis is accompanied by a rash of discoid erythematosus above it.

### 4.14 International normalized ratio of prothrombin time

Prothrombin time (PT) is the time until clotting of plasma due to the addition of a mixture of tissue thromboplastin and calcium and is a test primarily of extrinsic coagulation function. To eliminate interfacility variability, the World Health Organization proposed an international normalized ratio (INR). The PT‐INR is often used to evaluate the effectiveness of warfarin therapy. Higher values indicate that blood is less likely to coagulate and bleeding is more likely to occur.

### 4.15 Livedo reticularis

Livedo reticularis presents as reddish purple reticular macules as a symptom resulting from peripheral circulatory disorder of the skin. Related conditions include cutis marmorata, livedo reticularis, and livedo racemosa. Cutis marmorata is transient, appearing when the skin is exposed to cold outdoor air and disappearing when it is warmed, and can be considered a physiological phenomenon. Livedo reticularis and livedo racemosa are persistent conditions caused by organic changes in blood vessels. The rings of the retiform pattern are closed in livedo reticularis and open in livedo racemosa.

### 4.16 Antiphospholipid antibody

Commonly measured antiphospholipid antibodies include: (i) IgG‐ or IgM‐type anticardiolipin antibodies; (ii) IgG‐ or IgM‐type cardiolipin‐dependent anti–β2‐glycoprotein I antibodies; and (iii) lupus anticoagulants, which are immunoglobins that do not inhibit the activities of individual clotting factors but phospholipid‐dependently inhibit the blood‐clotting reaction. This reaction is measured by the activated partial thromboplastin time, diluted Russell viper venom time test, and platelet neutralization test, among others, and is detected by a method according to the Lupus Anticoagulant Guidelines of the International Society on Thrombosis and Hemostasis. When one of these is detected two or more times at a 12‐week interval, the condition is classified as APS. Phosphatidylserine‐dependent antiprothrombin antibody is attracting attention as a new antiphospholipid antibody. This antibody is correlated with the clinical symptoms of APS and the presence of lupus anticoagulants and can be measured in the serum, while the measurement of lupus anticoagulants requires high‐quality plasma.

### 4.17 Venous thrombosis

Thrombosis in a vein that involves a coagulation reaction in its pathogenic mechanism. The condition is caused by fibrin thrombi containing a large number of erythrocytes. Skin ulcers caused by venous thrombosis are typically shallow and have indistinct margins because they are caused by hypoxia of the skin tissue due to venous stasis. These ulcer often cause bleeding from the ulcer floor due to congestion.

### 4.18 Arterial thrombosis

Thrombosis occurring in an artery. Platelet coagulation is involved in its pathogenic mechanism. Skin ulcers due to arterial thrombosis are deep and have distinct margins, and bleeding from the ulcer floor is often unremarkable because they are caused by ischemia of the skin tissue supplied by the affected artery.

### 4.19 Erosion

A cutaneous or mucosal defect not extending beyond the basement membrane (dermoepidermal junction, mucosa). Usually resolves without leaving a scar.

### 4.20 Ulcer

A cutaneous or mucosal defect extending beyond the basement membrane (dermoepidermal junction, mucosa). Usually leaves a scar upon healing.

### 4.21 Gangrene

A condition in which the skin/subcutaneous tissue is necrotic and develops irreversible changes due to ischemia.

## 5 CHAPTER 5: EXPLANATION

### 5.1 Explanation 1: Effects of calcium antagonists, antiplatelet drugs, and prostaglandin preparations on SSc‐associated skin ulcers

#### 5.1.1 No study has evaluated the usefulness of calcium antagonists for treating SSc‐associated skin ulcers. However, calcium antagonists are useful for treating Raynaud phenomenon, and may be effective for circulatory disorder–associated ulcers

- As reports on the usefulness of nifedipine, Finch et al.^35^ performed a randomized crossover study of 16 SSc patients, and reported that nifedipine significantly reduced the frequency, duration, and severity of Raynaud phenomenon in comparison with a placebo. Meyrick Thomas et al.^36^ performed a randomized crossover study of 18 SSc patients, and found a significant decrease in the duration of Raynaud phenomenon. Thompson et al.^30^ performed a meta‐analysis regarding the effects of calcium antagonists on Raynaud phenomenon in SSc patients, and reported that nifedipine had been administered to a total of 44 SSc patients for 2 to 12 weeks in five studies, and that nifedipine significantly reduced the frequency, duration, and severity of Raynaud phenomenon in comparison with a placebo. A review of nicardipine did not show any significant difference in comparison with a placebo. This may have been due to the small number of patients (*n* = 15).

#### 5.1.2 No study has evaluated the usefulness of beraprost sodium for treating SSc‐associated skin ulcers, but it is useful for treating Raynaud phenomenon. This drug may be effective for circulatory disorder–associated ulcers

- Hida et al.^115^ administered beraprost sodium to 15 patients with SSc, three with mixed connective tissue disease, and one with Raynaud disease, and reported that this drug significantly decreased the frequency and duration of Raynaud phenomenon. Asano et al.^116^ investigated 12 SSc patients, and found that a sustained‐release beraprost sodium preparation significantly reduced the Raynaud condition score and symptoms. On the other hand, Vayssairat et al.^28^ performed an RCT of 107 SSc patients, and reported that beraprost sodium did not exhibit any significant inhibitory effects on Raynaud phenomenon or digital ulcers in comparison with a placebo, whereas it slightly delayed the appearance of digital ulcers in the winter, significantly improving overall well‐being.

#### 5.1.3 Intravenous prostaglandin preparations (such as alprostadil) may be effective for SSc‐associated digital ulcers

- Bartolone et al.^117^ performed an RCT of continuous alprostadil administration for 6 days in 12 SSc patients with serious Raynaud phenomenon. They reported an improvement in finger blood flow only in the treatment group after administration, along with reductions in the number, frequency, and severity of Raynaud phenomenon only in the treatment group. Gardinali et al.^118^ administered alprostadil to 36 SSc patients for 6 weeks (5 consecutive days per week) during the winter, and examined the healing of Raynaud phenomenon and digital ulcers. Alprostadil significantly decreased the frequency of Raynaud phenomenon, reducing the grade of Raynaud phenomenon. It was reported that complete healing was achieved in 12 of 14 patients with digital ulcers after alprostadil administration.
- Some RCTs were performed on intravenous iloprost injection in Europe and the United States. Tingey et al.^19^ conducted a meta‐analysis and found that intravenous injection of iloprost significantly reduced the frequency and severity of Raynaud phenomenon, significantly promoting digital ulcer healing. In Japan, iloprost inhalants for pulmonary arterial hypertension are covered by health insurance, but intravenous drugs are not commercially available.

### 5.2 Explanation 2: Surgical treatment for SSc‐associated skin ulcers

#### 5.2.1 Surgical treatment, such as excessive local debridement, for SSc‐associated refractory skin ulcers may further increase ulcer size, and caution is needed. On the other hand, surgical treatment is selected in some patients in whom conservative treatment does not relieve ulcers

- Debridement and split‐thickness skin grafting for SSc‐associated skin ulcers was studied in one case series study.^119^ Debridement induced finger shortening. After skin grafting, wound pain remained in some patients.
- One systematic review^120^ and one case series study^121^ investigated sympathectomy for SSc‐associated skin ulcers and short‐term therapeutic effects after sympathectomy were observed, but ulcer recrudescence and a delay in wound healing were noted. The long‐term effects of treatment remain to be clarified.
- One systematic review^56^ and one case series study^122^ examined joint contracture surgery for SSc‐associated skin ulcers at articular sites. Arthrodesis for flexion contracture of the PIP joint was shown to be effective, but surgical treatment for hyperextension of the MP joint was ineffective. In particular, for surgical treatment of serious contracture, such as finger‐in‐palm deformity, ostectomy was required The condition should be carefully managed from the viewpoint of finger shortening or cosmetic results.
- There is one case series study on the combination of bone marrow–exposing occlusive dressing and suction blister epidermal grafting for SSc‐associated skin ulcers.^123^ There was no significant difference in the interval until wound healing in comparison with standard treatment. However, this procedure may be a treatment option to minimize finger shortening in patients in whom bone exposure makes conservative treatment difficult.
- These studies were performed with the assumption that conservative treatment may initially be performed. However, it is also known that there are skin ulcers that may not respond to conservative treatment. When performing surgery for SSc‐associated skin ulcers, it should not be promptly performed, but procedures, such as split‐thickness skin grafting, may be attempted after achieving an improvement in the wound state by conservative treatment, considering the patient's QOL. In such patients, minimally invasive surgical treatment should be initially considered so that the affected finger may be preserved.

#### 5.2.2 Digital amputation for SSc‐associated skin ulcers lead to complications such as finger shortening and stump ulcers. Furthermore, of SSc‐associated skin ulcers may be repeatedly observed. For this reason, digital amputation should not be readily performed, except in unavoidable cases

- There is one case series study on digital amputation for SSc‐associated skin ulcers.^56^
- There are reports of digital amputation for SSc‐associated skin ulcers with advanced gangrenes, osteomyelitis, or purulent arthritis.^122^, ^124^ Surgical amputation for gangrenes may induce new stump ulcers, requiring additional surgical amputation. In patients with gangrene, spontaneous falling is the most appropriate for finger preservation.
- According to a case series study performed by Bogoch et al.,^56^ an average of 45% of patients with SSc had experienced digital ulcers during the course of their disease. Although the healing of these ulcers is often delayed and dry gangrene is frequent, spontaneous falling of the ulcer is considered the first choice from the viewpoint of finger shortening prevention. On the other hand, surgical amputation is primarily indicated for moist gangrene and osteomyelitis‐related articular destruction, which are difficult to relieve by conservative treatment. However, in such cases, minimally invasive surgical treatment should be initially considered before amputation so that fingers/toes may be preserved and the risk of stump ulcers after amputation be avoided.

### 5.3 Explanation 3: Testing and treatment for SLE‐related skin ulcers and oral ulcers

#### 5.3.1 If blister formation is observed in patients with SLE, direct/indirect immunofluorescence, histopathological examination, and examination for infectious diseases should be performed for differential diagnosis, considering various conditions including bullous lupus erythematosus

- In patients with SLE, bullous rash is relatively rare. Based on its pathogenesis, it is classified into three types: type 1 involves a rash in which marked liquefaction degeneration progresses to blister formation; type 2 consists of a rash complicated by other bullous diseases; and type 3 features a rash with histological findings similar to those of Duhring dermatitis herpetiformis, in which anti–type VII collagen autoantibody is typically present. Type 3 corresponds to narrow‐sense bullous lupus erythematosus. The diagnostic criteria prepared by Camisa et al.^125^ include: (i) the diagnostic criteria for SLE established by the American College of Rheumatology are met; (ii) blister formation in both sun‐exposed and nonexposed areas; (iii) histopathologically, the condition resembles Duhring dermatitis herpetiformis; (iv) antibasement membrane circulating antibody–negative or –positive reactions on indirect immunofluorescence with human skin samples; and (v) basement membrane IgG/IgA/IgM deposition is observed on direct immunofluorescence.
- Thus, detailed histopathological examination, including the fluorescent antibody method, is necessary for differentiation from diseases such as autoimmune bullosa. The fluorescent antibody method is not available in all institutions (direct immunofluorescence [outsourced examination is possible] for bullosa is covered by health insurance), but even only pathological examination should be performed. When differentiation is difficult, immunoelectron microscopy is necessary in some cases.^126^, ^127^
- Many patients with SLE are in an immunosuppressive state, and various infectious diseases may concomitantly occur. The possibility of various infectious diseases, including herpesvirus infection and contagious impetigo, other than primary disease–related conditions, should be considered, and microbiological testing is also necessary.
- Bullous rash often appears on SLE deterioration or in the presence of concomitant nephritis; caution is needed. Treatments include systemic steroid administration and hydroxychloroquine therapy. The usefulness of hydroxychloroquine for treating cutaneous/SLE was reported in Japan.^128^
- Many studies have reported the effectiveness of DDS.^126^, ^129^, ^130^ In this case, low‐dose (25–50 mg/day) DDS administration resulted in the loss of new blisters in 24 to 48 h, leading to the disappearance of rash in 7 to 10 days. However, for the use of DDS, the risk of drug eruption in patients with SLE should be considered.

#### 5.3.2 Etiological factors for SLE‐related leg ulcers include vasculitis, thrombosis including antiphospholipid syndrome, circulatory disorder such as venous ulcers and peripheral arterial obstruction, infectious disease, and pyoderma gangrenosum. To investigate the etiology, hematology, skin biopsy, culture tests for causative bacteria, testing for venous insufficiency/thrombosis using lower limbs venous ultrasound, and testing for limb ischemia with the ankle pressure, ankle brachial pressure index, and skin perfusion pressure should be performed

- Chia et al.^131^ examined 29 patients with connective tissue disease with leg ulcers, including eight with SLE. The most frequent etiological factor was “venous ulcer” (34.5%), followed by multifactorial ulcers (24.1%), vasculitis (20.7%), nontuberculous mycobacteriosis (7.0%), pyoderma gangrenosum (7.0%), peripheral arterial obstruction (3.4%), and iatrogenic ulcers (3.4%). Dabiri et al.^132^ performed a case series study, and reported that the frequent sites of leg ulcers in patients with SLE were the medial and lateral malleoli, upper areas of the medial and lateral malleoli, and anterior tibial region, and that etiological factors included vasculitis, venous insufficiency, thrombosis (including antiphospholipid syndrome), lupus erythematosus profundus, and concomitant lichen planus. They found that local treatment at the ulcer site was insufficient in many cases, suggesting the importance of primary disease treatment.
- Based on the above findings, skin biopsy, infectious disease testing by culture tests, and detailed examination for circulatory disorder may be necessary for differentiating the pathogenesis of leg ulcers in patients with SLE (with respect to testing for venous leg ulcers, refer to guidelines for the management of lower leg ulcers/varicose veins). For the differentiation of antiphospholipid syndrome, anticardiolipin IgG/IgM antibodies, lupus anticoagulant, and β2‐glycoprotien I‐dependent anti‐CL IgG/IgM antibodies are covered by health insurance in Japan.
- Minimally invasive examinations for limb ischemia related to arterial obstruction include measurement of the ankle pressure, ankle brachial pressure index, and skin perfusion pressure. If these tests indicate an abnormality, magnetic resonance angiography, computed tomography angiography, and angiography should be performed (with respect to the diagnosis/treatment of arterial obstruction–related ulcers, refer to guidelines for the management of diabetic ulcers and gangrene).

#### 5.3.3 Many SLE‐associated oral ulcers are painless, being related to a primary disease. However, there are some cases of discoid lupus erythematosus of the mucosa. Diseases to be differentiated include recurrent aphthous stomatitis, lichen planus, leukoplakia, and infectious diseases such as herpes simplex and fungal infection. When it is difficult to make a diagnosis based on clinical symptoms, skin biopsy, including direct immunofluorescence, and investigation for infectious disease are recommended for detailed examination for the etiology. Furthermore, SLE‐associated oral ulcers may reflect the disease state, and it should be re‐evaluated using blood biochemistry/immunological examination and urinalysis

- In patients with SLE, the prevalence of oral lesions varies among reports, ranging from 2% or 3% to 50%.^133^ The lesion site is painless in some cases but painful in others. The ulcer site sometimes becomes painful, affecting lifestyle. Typical oral lesions include painless ulcers, which often occur in the upper palate in the acute stage, and discoid lupus erythematosus of the mucosa. However, various disease types, such as recurrent aphthous stomatitis, lichen planus‐like exanthema, leukoplakia, and red plaque, have been reported.^133^
- In patients with SLE receiving systemic administration of immunosuppressants, secondary infection with herpes virus or fungus may induce oral ulcers^133^; therefore, microscopy, Tzanck test, and viral antigen tests must be performed. If herpetic gingivostomatitis or oral candidiasis is observed, treatment with antiviral or antifungal drugs should be performed.
- Immunoglobulin deposition in the basement membrane is observed on direct immunofluorescence in ≥70% of patients with SLE‐related oral lesions, whereas the positive rate in patients with lichen planus–like exanthema or leukoplakia is ≤10%. It is useful for the differential diagnosis.^134^ In the case of refractory ulcers at the site of discoid lupus erythematosus, the possibility of scar carcinoma must also be considered.
- Oral lesions are adopted for many criteria as a parameter of SLE activity.^135^, ^136^, ^137^ Therefore, if oral lesions are observed in patients with SLE, SLE activity should be initially evaluated, and the systemic administration of steroids or immunosuppressants should be performed if necessary. The systemic administration of steroids or immunosuppressants is also useful for treating oral lesions.
- Systemic treatment for SLE‐associated oral ulcers is beneficial, but no study has directly evaluated the utility of local therapy. However, there is one systematic review on local treatment for recurrent aphthous stomatitis.^133^ Topical steroid therapy is the first choice of therapy.

#### 5.3.4 As lupus erythematosus profundus may promote skin ulcer/scar/excavation formation with progression, early therapeutic intervention is necessary. Systemic steroid administration, hydroxychloroquine, and DDS are treatment options

- Generally, treatments for SLE‐associated skin lesions include topical steroids, hydroxychloroquine, DDS, thalidomide, immunosuppressants, oral clofazimine (as an anti‐Hansen disease drug), and systemic steroid administration.^138^
- The initial dose of a steroid for lupus erythematosus profundus varies, depending on the state of SLE or properties of exanthema. One study reported an administration of 5 to 60 mg/day of prednisolone,^139^ and another study reported a dose range of 3 to 25 mg/day (mean: 10.8 mg/day).^140^ However, to minimize the appearance of skin ulcers and scars/excavation, prednisolone administration at a reference dose of 0.5 mg/kg per day may be appropriate.
- One systematic review^141^ assessed the efficacy of hydroxychloroquine for SLE. Park et al.^142^ administered hydroxychloroquine to 15 of 17 patients with lupus erythematosus profundus, and reported that 58.9% of the patients responded to hydroxychloroquine alone.
- Ujiie et al.^143^ summarized 10 DDS‐treated patients with lupus erythematosus profundus. DDS administration at 25 to 75 mg/day relieved exanthema, with a mean interval of approximately 4.6 weeks. Thus, DDS administration is also an option, but the use of DDS induces a high incidence of drug eruption in SLE patients; caution is needed.

### 5.4 Explanation 4: Examination and treatment for dermatomyositis‐associated skin ulcers

#### 5.4.1 Dermatomyositis‐associated skin ulcers occur by various mechanisms of pathogenesis: primary disease–related ulcers to ulcers related to secondary factors such as infection. To evaluate the pathogenesis, skin biopsy and microbiology tests for bacteria/fungus/acid‐fast bacteria should be performed

- The incidence of dermatomyositis‐associated skin ulcers is 3% to 19% according to several case series studies.^144^, ^145^, ^146^ There are various etiologies of dermatomyositis‐associated skin ulcers: primary disease–related ulcers to secondary factor–related ulcers. Primary factors include vasculopathy, vasculitis, excessive infiltration of inflammatory cells at the dermoepidermal junction, infectious disease, and erosion associated with pruritus‐related scratching behavior. Therefore, it is recommended to perform skin biopsy and microbiology tests for bacteria/fungus/acid‐fast bacteria and closely investigate the etiology of ulcers.
- Yamasaki et al.^147^ performed case series analysis and extracted “skin ulcer” as an independent risk factor for the prognosis of dermatomyositis.

#### 5.4.2 If findings suggestive of vascular damage, such as purpura and necrosis, at the dermatomyositis‐associated skin ulcer site are observed, imaging procedures, blood gas analysis, pulmonary function testing, and blood testing should be performed to evaluate the presence or absence of concomitant interstitial lung disease

- In dermatomyositis patients with skin ulcers suggestive of vascular damage, such as purpura and necrosis, the incidence of interstitial lung disease may be high. Especially when skin ulcers are present in patients with CADM, it is important to consider rapidly progressive interstitial lung disease and regularly perform imaging procedures, blood gas analysis, and blood testing for early diagnosis.
- Among CADM patients with pulmonary lesions, a group with the rapid progression of pulmonary lesions was compared with a group with the slow progression of pulmonary lesions. In the former, skin ulcers were observed in seven (78%) of nine patients, and the percentage was higher than in the latter (three of nine [33%]).^148^
- Hamaguchi et al.^149^ analyzed 77 patients with dermatomyositis and reported that skin ulcers were observed in 30 (70%) of 43 patients positive for anti‐MDA5 antibody, which is frequently detected in patients with CADM, and in four (12%) of 34 patients positive for other autoantibodies. Similarly, Cao et al.^150^ found that skin ulcers were present in 12 (80%) of 15 anti‐MDA5 antibody‐positive dermatomyositis patients and in four (8.2%) of 49 anti‐MDA5 antibody‐negative dermatomyositis patients. Koga et al.^151^ reported that skin ulcers were noted in 10 (59%) of 17 anti‐MDA5 antibody‐positive dermatomyositis patients and in seven (12%) of 62 anti‐MDA5 antibody‐negative dermatomyositis patients. Therefore, a myositis‐specific antibody and anti‐ARS antibody, as well as anti‐MDA5 antibody, anti–TIF‐1γ antibody, and anti–Mi‐2 antibody if possible, should be tested.
- Ishigaki et al.^152^ investigated 39 patients with dermatomyositis and reported that skin ulcers were frequent in patients with acute/subacute interstitial pneumonia and that the prognosis was significantly poorer than in those without skin ulcers.

#### 5.4.3 Dermatomyositis‐related calcinosis cutis may cause skin ulcers, and imaging procedures and endocrinological examination should be performed for detailed examination of the etiology

- Cutaneous or soft tissue calcinosis is often observed in patients with dermatomyositis. In particular, its incidence is approximately 40% in patients with childhood dermatomyositis. Although the incidence is low in patients with SLE, ectopic calcinosis is sometimes observed.^153^ Calcinosis may occur in all sites: skin/soft tissue, vertebral, and costal regions.^154^ In patients with childhood dermatomyositis, the incidence of muscular/subcutaneous calcinosis is higher than in adults.^155^
- In the classification of calcinosis cutis,^156^ calcinosis in patients with dermatomyositis or SLE corresponds to dystrophic calcification. The mechanism of tissue calcinosis remains to be clarified, but etiological factors include tissue/vascular damage, ischemia, and age‐related tissue changes. In addition, tissue calcinosis may develop when there is a decrease in calcification‐inhibiting factors or when a substance that promotes calcification as a crystal nucleus appears. Furthermore, it was reported that the levels of calcium‐binding amino acids and γ‐carboxyglutamic acid in the patient tissue with calcinosis were increased, and that there was an increase in the urinary γ‐carboxyglutamic acid level.^157^
- To differentiate the above pathogeneses involved in cutaneous/soft tissue calcinosis, it is necessary to measure blood calcium/phosphorus and parathyroid hormone (PTH) levels. However, recent studies have shown that acroosteolysis and digital ulcers in patients with SSc were positively correlated with subcutaneous calcinosis.^158^, ^159^ Furthermore, another study performed a correlation between calcinosis and PTH.^160^ Caution is needed for interpreting PTH levels.
- Blane et al.^161^ classified childhood dermatomyositis–related calcinosis into four types based on x‐ray findings: I: deep linear calcinosis, II: deep mass‐like calcinosis, III: subcutaneous superficial‐layer mass‐like calcinosis, and IV: subcutaneous net‐like lacy calcinosis.
- Calcinosis is incidentally detected on x‐ray findings in many cases. When palpation reveals subcutaneous induration, plain x‐ray or computed tomography for confirming its properties is useful.^156^

#### 5.4.4 Panniculitis in patients with dermatomyositis reflects the disease state and may cause scars/ulcers; therefore, systemic steroid administration is selected for treatment. If there is no response to steroid treatment, the systemic administration of immunosuppressants, such as cyclosporine, methotrexate, and azathioprine, should be considered

- Panniculitis is reportedly observed in approximately 9% of patients with dermatomyositis. Douvoyiannis et al.^162^ investigated 24 dermatomyositis patients with panniculitis in the United Kingdom and reported that frequent sites were the gluteal region, femur, and arms. Histopathologically, lobular panniculitis is typically noted, primarily consisting of lymphocyte infiltration. Several studies have shown that concomitant diseases, such as interstitial lung disease, were frequent in the presence of membranocystic changes, suggesting that these changes contribute to a poor prognosis.^163^, ^164^ Furthermore, membranocystic changes in the adipose tissue and interstitial lung disease may be pathologically associated with microvascular damage.^165^
- For treatment, systemic steroid administration or dose elevation is attempted in many cases, and the responsiveness to treatment is relatively favorable.^162^, ^166^ According to a report by Douvoyiannis et al.,^162^ an improvement in exanthema was achieved in 89% (eight of nine) of patients treated with steroids alone. A rapid reduction in the steroid dose resulted in the deterioration of panniculitis or dermatomyositis in two patients. However, in these patients, additional steroid dose elevation led to an improvement. The dose of steroids ranged from 0.3 to 2 mg/kg per day.
- According to a summary on previous case reports in Japan published by Fujisawa et al.,^166^ prednisolone at 10 to 60 mg/day had been administered. Of the patients, 69% (11 of 16) responded to this therapy, whereas 31% (five of 16) did not respond, including patients who died. The nonresponders included patients with membranocystic changes and those with interstitial lung disease. Attention should be paid to membranocystic changes in the adipose tissue.
- Oral immunosuppressants are also used in combination with oral steroids. As the type of immunosuppressants, cyclosporine (≤3 mg/kg per day), methotrexate (2.5–7.5 mg/week), and azathioprine (2–3 mg/kg per day) are combined with steroids.^162^
- Furthermore, there are case reports in which intravenous immunoglobulin led to dramatic improvements in two patients who did not respond to these immunosuppressive therapies.^167^, ^168^
- Thus, systemic steroid administration (dose elevation) should be initially considered for panniculitis in patients with dermatomyositis. Immunosuppressants should be administered based on the treatment responsiveness.

### 5.5 Explanation 5: Treatment for vasculitis‐associated skin ulcers

#### 5.5.1 To treat vasculitis‐associated skin ulcers, conservative treatment should be prioritized. As there is no sufficient evidence on the usefulness of surgical treatment including osteotomy/disarticulation, it should not be readily performed

- For skin ulcers that may not be cured by conservative treatment, minimally invasive surgical treatment should be initially considered. One study performed pinch skin grafting in patients with rheumatoid vasculitis, and the number of patients with relief was small.^169^
- Another study adopted a combination of bone marrow–exposing occlusive dressing and suction blister epidermal grafting in vasculitis patients with refractory skin ulcers, but there was no response, ultimately requiring osteotomy.^170^
- According to another study, epithelization was achieved in most patients by continuing conservative treatment for vasculitis‐related ulcers.^171^ On the other hand, the recurrence rate of vasculitis‐related ulcers is high, and there are few cases in which surgical treatment is indicated.
- No study has reported that the results of surgical treatment for vasculitis‐related ulcers are better than those of conservative treatment. Surgical treatment cannot be recommended. If extensive gangrene requires osteotomy/disarticulation, whether these are indicated should be carefully examined after sufficiently evaluating the state of vasculitis and blood flow.

### 5.6 Explanation 6: Diagnosis and treatment of RA‐associated skin ulcers

#### 5.6.1 There are many reports on RA suggesting the onset/deterioration of rheumatoid vasculitis during therapy with TNF‐α inhibitors. In patients in whom the causal relationship is strongly suspected, TNF‐α inhibitors should be discontinued, and a switch to other drugs should be considered

- A cohort study^172^ on the relationship between the onset of rheumatoid vasculitis and TNF‐α inhibitors was conducted and showed that insufficient treatment for a primary disease may be involved in the onset/deterioration of rheumatoid vasculitis. In addition, there are patients in whom the use of TNF‐α inhibitors is suggested. In those with RA in whom the onset/deterioration of vasculitis is suspected, the presence or absence of TNF‐α inhibitor therapy should be investigated. If the involvement of this drug in the onset/deterioration is strongly suspected, the use of this agent should be discontinued, and a switch to other drugs should be considered.
- With the recent widespread use of TNF‐α inhibitors, their usefulness for treating many autoimmune diseases, including RA, has been established. On the other hand, many reports on adverse reactions to these drugs have been accumulating. Primary adverse reactions include severe infectious disease, opportunistic infection such as tuberculosis, demyelinating disease, and lymphoproliferative disease. However, recently, patients who developed autoimmune diseases, such as vasculitis, SLE, and interstitial lung disease, after the use of these drugs have been increasingly reported.
- In 1999, Brion et al.^173^ reported the first patient who developed rheumatoid vasculitis after the use of a TNF‐α inhibitor. Since then, similar reports^174^, ^175^, ^176^, ^177^, ^178^, ^179^ have been successively presented, including a patient in whom the use of a TNF‐α inhibitor worsened their rheumatoid vasculitis,^180^ a patient who developed optic neuropathy,^181^ a patient who developed glomerulonephritis,^182^ and patients who developed ANCA‐associated vasculitis.^183^, ^184^ Ramos‐Casals et al.^185^ investigated 226 patients with autoimmune diseases after the use of TNF‐α inhibitors (187 with RA, 17 with Crohn disease, seven with ankylosing spondylitis, six with psoriatic arthritis, five with juvenile RA, three with other conditions), and reported the onset of vasculitis in 113 patients (95 with RA). Histopathologically, leukocytoclastic vasculitis accounted for 63%, necrotic vasculitis for 17%, and lymphocytic vasculitis for 6% of cases. Findings of leukocytoclastic vasculitis are most commonly observed.
- Concerning the incidence of vasculitis, Flendrie et al.^172^ performed a cohort study, and reported an incidence of 3.9%. The relationship between the use of TNF‐α inhibitors and onset of vasculitis remains to be clarified. However, their causal relationship is strongly suspected for the following reasons: (i) the timing of vasculitis onset is consistent with the start of treatment with TNF‐α inhibitors; (ii) exanthema at the site of injection initially occurred and enlarged to the whole body in some RA patients who developed vasculitis after etanercept use; (iii) vasculitis was relieved in ≥90% of patients after the discontinuation of TNF‐α inhibitors; (iv) the additional administration of TNF‐α inhibitors led to recurrent exanthema/recrudescence in 75% of patients; and (v) vasculitis occurs even in the presence of diseases of which the pathogeneses do not involve vasculitis, such as psoriasis.

#### 5.6.2 For the treatment of RA‐associated skin ulcers, the use of peripheral circulation‐improving drugs/antiplatelet drugs, such as argatroban hydrate, alprostadil, sarpogrelate hydrochloride, cilostazol, and beraprost sodium, should be considered

- Chen et al.^186^ reported that various blood vessels: dermal venules to adipose tissue arterioles were damaged in patients with rheumatoid vasculitis, and that degeneration of various blood vessels (small/medium/large) was observed even in clinically healthy skin. Furthermore, Westedt et al.^187^ and Fitzgerald et al.^188^ reported that vasculitis was histopathologically observed even in apparently healthy skin in approximately 30% of patients with RA. Such abnormalities may cause circulatory disturbance in the phase of ulcer healing regardless of vasculitis‐related and nonvasculitis‐related conditions, hindering wound healing. Peripheral circulation‐improving drugs/antiplatelet drugs that are routinely used to treat skin ulcers associated with other connective tissue diseases may be useful. Therefore, no study has reported their effectiveness for treating skin ulcers in patients with RA, but argatroban hydrate injection, alprostadil injection, and oral peripheral circulation‐improving drugs/antiplatelet drugs, such as sarpogrelate hydrochloride, cilostazol, and beraprost sodium, may also be used for the treatment of skin ulcers in patients with RA.

### 5.7 Explanation 7: Diagnosis and treatment of antiphospholipid syndrome–associated skin ulcers

#### 5.7.1 In patients with a history of skin ulcers related to antiphospholipid syndrome–associated venous thrombosis, warfarin may be effective in prevention. On the other hand, in those without such a history, warfarin remains an option for prevention

- For the secondary prevention of venous thrombosis, high‐dose warfarin therapy has been considered to be necessary based on case series studies.^189^, ^190^ However, according to follow‐up of 114 antiphospholipid antibody‐positive patients with a history of thrombosis, with a mean follow‐up of 2.7 years, the recurrence rate of thrombosis in patients treated at a high PT‐INR (3.1–4.0) was 10.7%, whereas that in those treated at a medium PT‐INR (2.0–3.0) was 3.4%.^191^ In addition, according to a follow‐up of 109 antiphospholipid antibody‐positive patients with a history of thrombosis, with a mean follow‐up of 3.6 years, the recurrence rate of thrombosis in patients treated at a high PT‐INR was 11.1%, whereas in those treated at a medium PT‐INR was 5.5%.^192^ A meta‐analysis^193^ of the two RCTs showed that there were no differences in the recurrence rate of thrombosis or frequency of serious hemorrhage between high and medium PT‐INR, and that the frequency of microhemorrhage significantly increased at a high PT‐INR. Therefore, we cannot conclude that a higher PT‐INR (≥3.0) is more effective than a medium PT‐INR (2.0–3.0), and the risk of hemorrhage rather increases; a medium PT‐INR (2.0–3.0) is recommended for patients with venous thrombosis.
- Concerning arterial thrombosis, a consensus regarding treatment has not been reached among recent studies. Warfarin was shown to be effective in preventing recurrent thrombosis, which was common among recent studies,^193^, ^194^, ^195^ but an optimal PT‐INR is unclear. These results were obtained in studies on Europeans and Americans, and there is no report on Japanese patients. Furthermore, no study has examined an optimal PT‐INR in Japanese patients.
- Concerning the therapeutic effects of direct oral anticoagulants (DOAC) on thromboembolism and their preventive effects on recurrence, there are three RCTs (investigational drug: rivaroxaban in all studies).^196^, ^197^, ^198^ Among APS patients with a history of venous thrombosis, the incidence of recurrent thrombosis in rivaroxaban‐treated patients was slightly higher than in warfarin‐treated patients. It was much higher in patients positive for all three antiphospholipid antibodies. According to the European League Against Rheumatism (EULAR) recommendations for the management of antiphospholipid syndrome in adults in 2019,^199^ the use of rivaroxaban should be avoided in patients with APS positive for all three antiphospholipid antibodies and that its application should be considered when the intensity of treatment (PT‐INR: 2.0–3.0) cannot be achieved despite the use of high‐dose vitamin K antagonists or when the use of vitamin K antagonists is contraindicated.

#### 5.7.2 For the prevention of antiphospholipid syndrome–associated skin ulcers, combination therapy with antiplatelet drugs, such as aspirin, ticlopidine hydrochloride, and dipyridamole, is an option for high‐risk groups with thrombosis and patients in whom warfarin alone results in recurrent thrombosis

- Previously, it was considered that low‐dose oral aspirin administration was effective in preventing thrombosis in patients without a history of thrombosis despite the continuous detection of antiphospholipid antibodies.^200^ However, in 2007, Erkan et al.^201^ denied the efficacy of low‐dose aspirin in primary prevention based on the results of a multicenter cooperative RCT. The participants included 98 continuously antiphospholipid antibody‐positive patients without a history of thrombosis/recurrent pregnancy loss. The incidence of acute thromboembolism during the 2‐ to 3‐year follow‐up was compared between low‐dose aspirin and placebo. There was no significant difference. Therefore, currently, there is no evidence on the usefulness of aspirin for the primary prevention of thrombosis. However, there is an opinion that primary prevention by aspirin should be considered in some patients by examining risk factors.^201^
- In patients in whom warfarin administration alone resulted in recurrent thrombosis‐associated skin ulcers, antiplatelet drugs, such as low‐dose aspirin, ticlopidine hydrochloride, and dipyridamole, were combined with warfarin, and there were responders. However, there is no evidence on the effectiveness of combination therapy.
- In addition, combination therapy with warfarin and antiplatelet drugs further increases the risk of hemorrhage, and it is necessary to carefully administer these drugs while reviewing clinical symptoms and laboratory data.

#### 5.7.3 For the prevention of antiphospholipid syndrome–associated skin ulcers, the permanent administration of anticoagulants is an option

- The characteristics of antiphospholipid syndrome–related thrombosis are as follows: it develops in arteries/veins and large/small/capillary blood vessels; and it recurs in 50% of patients within 6 months after the initial onset of thrombosis in the absence of treatment and in 80% within 2 years.^190^
- According to a non‐RCT with a 4‐year follow‐up of 34 anticardiolipin antibody‐positive patients,^202^ recurrent thrombosis was observed in 20% of patients in the warfarin‐discontinued group and in only 5% in the anticoagulant therapy–continued group, although there was no significant difference.
- Based on the above findings, permanent prophylactic administration may be necessary, but there is no evidence. However, if the bleeding tendency occurs in anticoagulant therapy–continued patients, the prognosis may be poor; caution is needed.

#### 5.7.4 For antiphospholipid syndrome–associated skin ulcers, multidisciplinary treatment, such as systemic steroids, plasmapheresis, and heparin, should be administered in patients with catastrophic antiphospholipid syndrome complicated by extensive skin necrosis or digital necrosis. In those with antiphospholipid syndrome other than the catastrophic form, treatment with anticoagulants, including warfarin, or antiplatelet drugs should be considered

- Most serious skin lesions, such as digital necrosis, extensive skin necrosis, and skin ulcers, are associated with arterial thrombosis. In patients with refractory skin ulcers or extensive skin necrosis, thrombosis of other organs may be present. It is necessary to evaluate thrombosis of the eye/brain/lung/heart/kidney/lower limb blood vessels. Furthermore, a study showed that potent anticoagulant therapy with heparin or low‐molecular‐weight heparin was effective for refractory skin ulcers/skin necrosis.^13^
- After evaluating thrombosis of other organs, as described above, treatment in accordance with arterial thrombosis may be attempted for serious skin lesions.
- For noncatastrophic antiphospholipid syndrome–associated skin ulcers, anticoagulants, including warfarin, are routinely administered, and combined with antiplatelet drugs in some patients.^203^
- After the acute stage of catastrophic antiphospholipid syndrome, the continuous secondary prevention of thrombosis by anticoagulant (primarily warfarin) therapy or combination therapy with antiplatelet drugs in some patients is necessary.
- In patients in whom there is no improvement in hypercoagulability despite warfarin therapy or combination therapy with antiplatelet drugs, intravenous immunoglobulin therapy^204^ and rituximab administration^205^ should be attempted to decrease the level of antiphospholipid antibody, which is considered to be an etiological factor.
- A noncontrolled, open‐label pilot study of rituximab was performed.^206^ The participants included 19 patients, and the efficacy and safety of rituximab were examined during a 1‐year period of observation. The improvement ratings for thrombopenia, valvular heart disease, skin ulcers, kidney damage, and neuropathy were evaluated. The results suggested that some symptoms, such as skin ulcers, are relieved, although rituximab does not reduce all symptoms of antiphospholipid syndrome. There was no significant finding concerning safety.
- In patients in whom skin ulcers remain after a warfarin‐ or combined antiplatelet drug–related improvement in hypercoagulability, surgical treatment, such as skin grafting, may also be attempted.

## 6 CHAPTER 6: DETAILS OF A SYSTEMATIC REVIEW ON EACH CQ

CQ1: Are there any drugs effective in preventing or treating SSc‐associated skin ulcers?

**Prevention**

**Table:** 

Recommendation level	Description of recommendation
Bosentan: strong recommendation PDE5 inhibitors: weak recommendation	The prophylactic administration of bosentan for SSc‐associated skin ulcers is recommended. The prophylactic administration of PDE5 inhibitors (sildenafil, tadalafil) for SSc‐associated skin ulcers is proposed

**Treatment**

**Table:** 

Recommendation level	Description of recommendation
Bosentan: weak recommendation PDE5 inhibitors: weak recommendation	Treatment with bosentan or PDE5 inhibitors (sildenafil, tadalafil) for SSc‐associated skin ulcers is proposed

### 6.1 Literature search

A request to the Japan Medical Library Association was combined with retrieval by manual search (refer to Supplement Materials with respect to the retrieval style and search results.)

Databases used: PubMed, Cochrane Library, Japanese Medical Abstracts Society.

Search period: January 1980 to December 2020.

Results: PubMed: 508 documents, Cochrane Library: 204, and Japanese Medical Abstracts Society: 57 were retrieved. A total of 654 documents were reviewed: 651, excluding database‐duplicated articles, and three were added from the references in the articles.

### 6.2 Outcome

As a result of consideration by the systematic review team, patients, including those with SSC (diffuse cutaneous SSc, limited cutaneous SSc), were randomly assigned, and two items were adopted: (i) prevention of new ulcer onset (risk of new ulcer onset, number of persons with new ulcer onset, mean number of new ulcers): importance of outcome 5, and (ii) healing of existing ulcers (number of ulcers, mean number of ulcers, number of persons with ulcer healing [improvement], number of cured [reduced] ulcers): importance of outcome 6. The importance of outcome was determined by all guideline‐drafting members' agreement (consistency rate for all imaging procedures: 100%).

### 6.3 Literature screening

**FIGURE 7:**
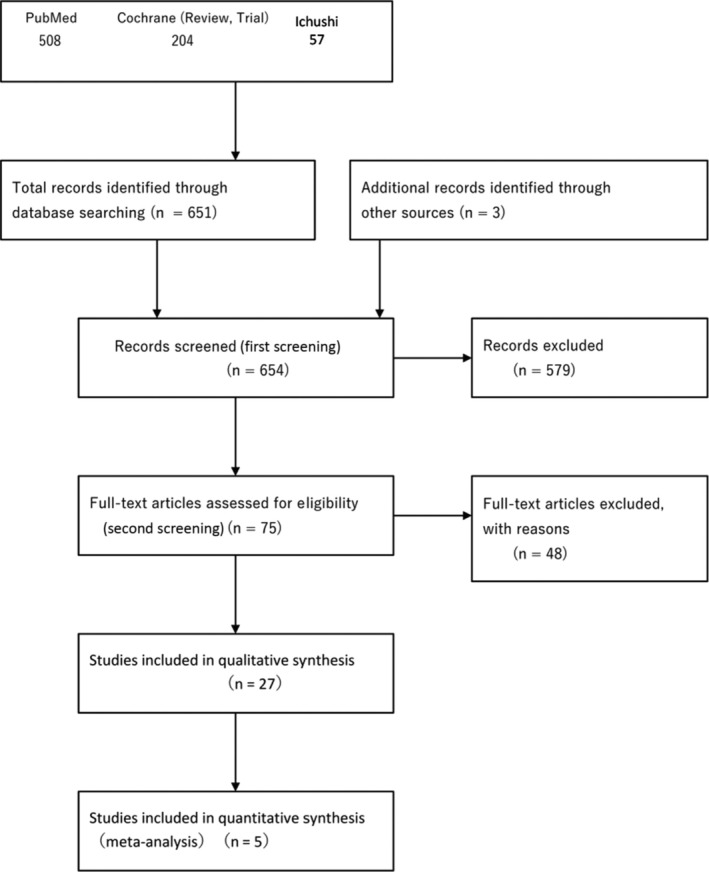
Clinical question 1 (CQ1): flow chart of the literature search.

Primary screening was performed, and 75 documents describing the above outcomes were selected. On secondary screening, only RCTs were selected, and 27 remained. Finally, articles in which there is a description on the healing of skin ulcers but with no relevant relationship were excluded, and the remaining five articles were used for analysis. A flow chart of the literature search is presented in Figure 7.

### 6.4 Evaluation of individual references

With respect to the 27 RCTs selected on secondary screening, the selection bias, performance bias, detection bias, attribution bias, and other biases were assessed based on the Minds Manual for Guideline Development 2020 version 3.0.

### 6.5 Evaluation of the outcome

Concerning PDE5 inhibitors, a meta‐analysis was performed with respect to two items: the healing of skin ulcers and the onset of new skin ulcers. For outcome assessment, the integrated value and 95% CIs of effect measures for the respective outcomes were calculated using Review Manager 5.4.

### 6.6 Results

Healing of skin ulcers: integrated value of effect measures 1.57 (95% CI, 1.14–2.17), strength of total evidence B (middle).

Development of new skin ulcers: integrated value of effect measures 0.44 (95% CI, 0.22–0.89), strength of total evidence B (middle).

**FIGURE 8:**
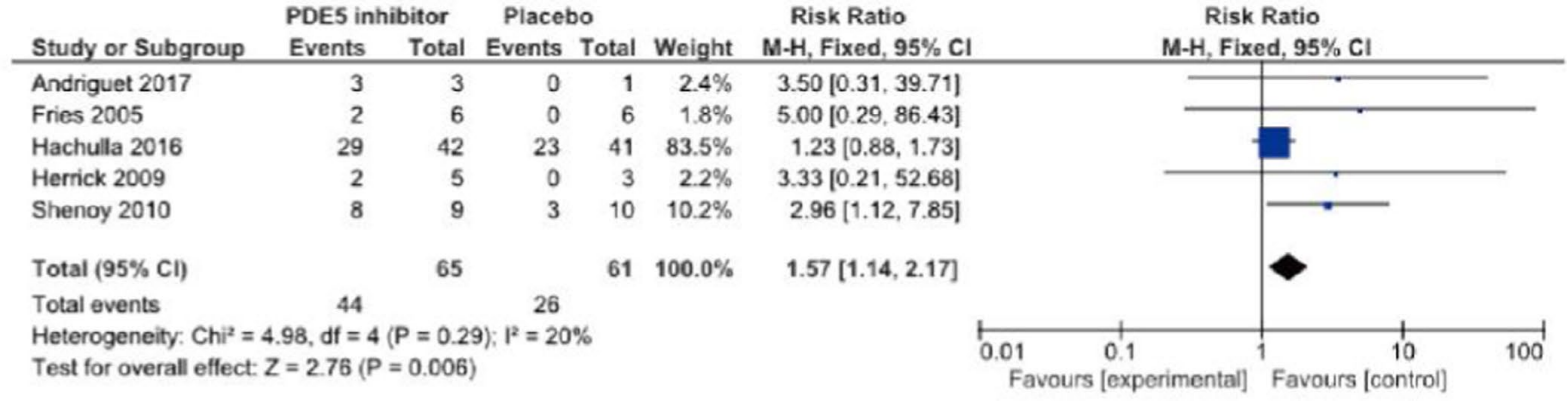
Clinical question 1 (CQ1): forest plot of PDE5 inhibitors (outcome: skin ulcer healing). CI, confidence interval; M‐H, Mantel‐Haenszel.

**FIGURE 9:**
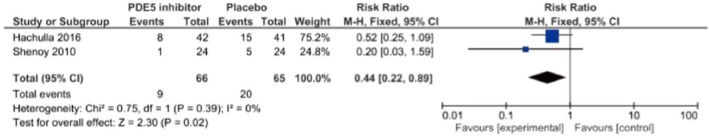
Clinical question 1 (CQ1): forest plot of PDE5 inhibitors (outcome: onset of new skin ulcers). CI, confidence interval; M‐H, Mantel‐Haenszel.

**FIGURE 10:**
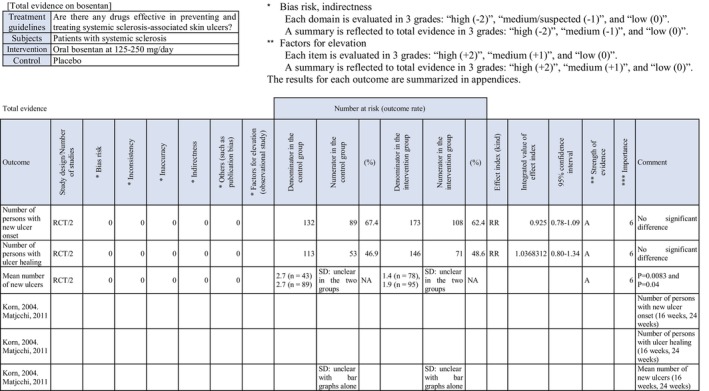
Clinical question 1 (CQ1): total evidence on bosentan. NA, not applicable.

**FIGURE 11:**
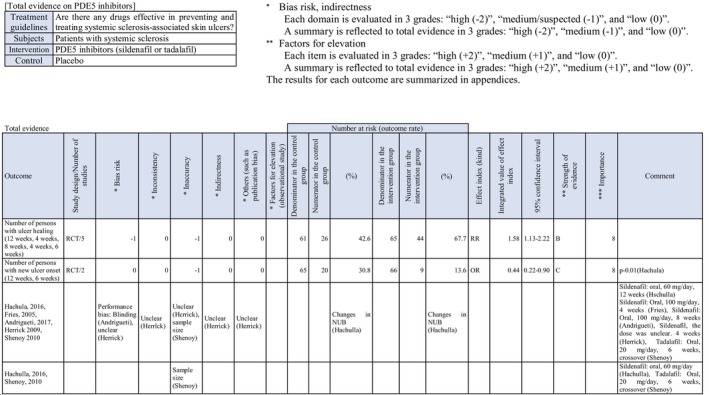
Clinical question 1 (CQ1): total evidence on PDE5 inhibitors.

A forest plot and total evidence are presented (Figures 8, 9, 10, 11) (with respect to assessment sheets, refer to Supplementary Materials).

**FIGURE 12:**
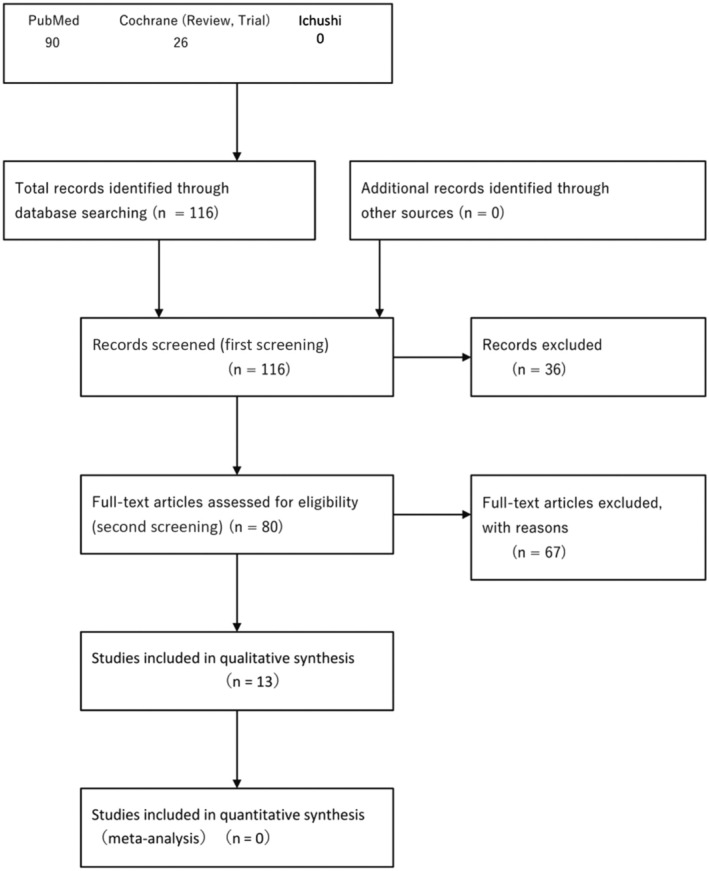
Clinical question 2 (CQ2): flow chart of the literature search.

**FIGURE 13:**
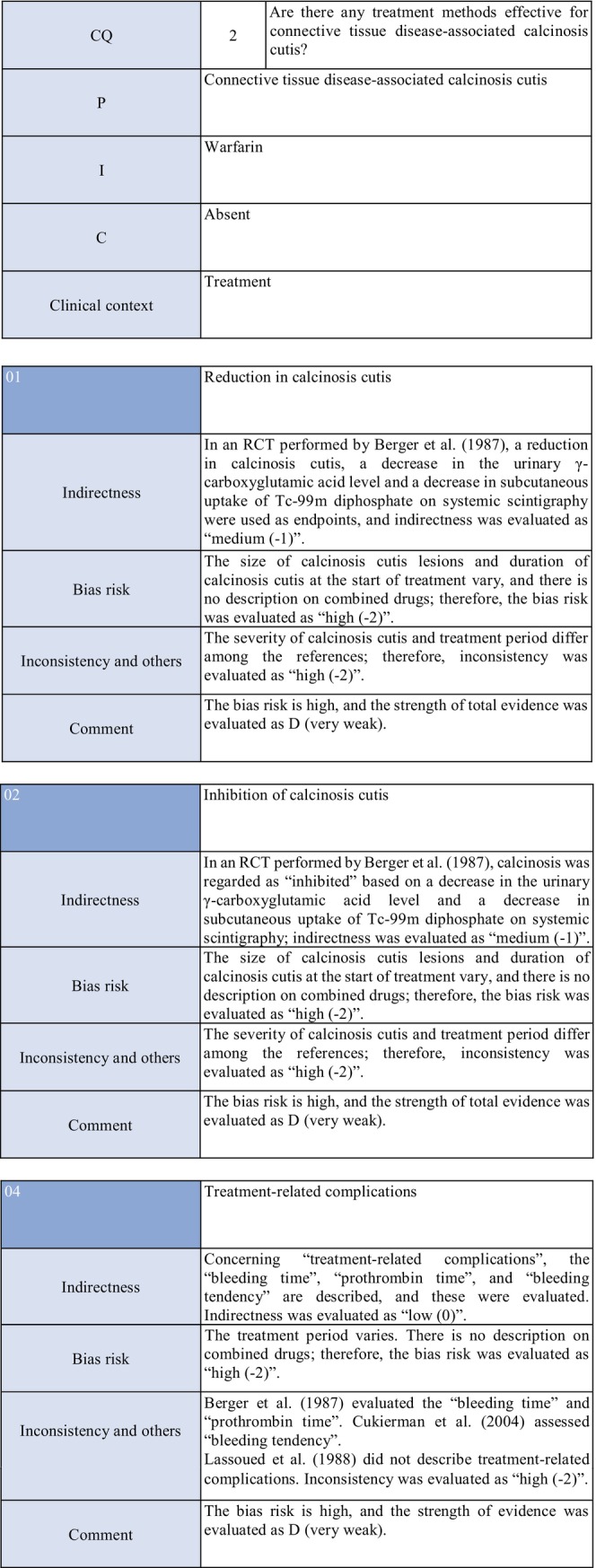
SR‐9: qualitative systematic review. CQ, clinical question; RCT, randomized controlled trial.

**FIGURE 14:**
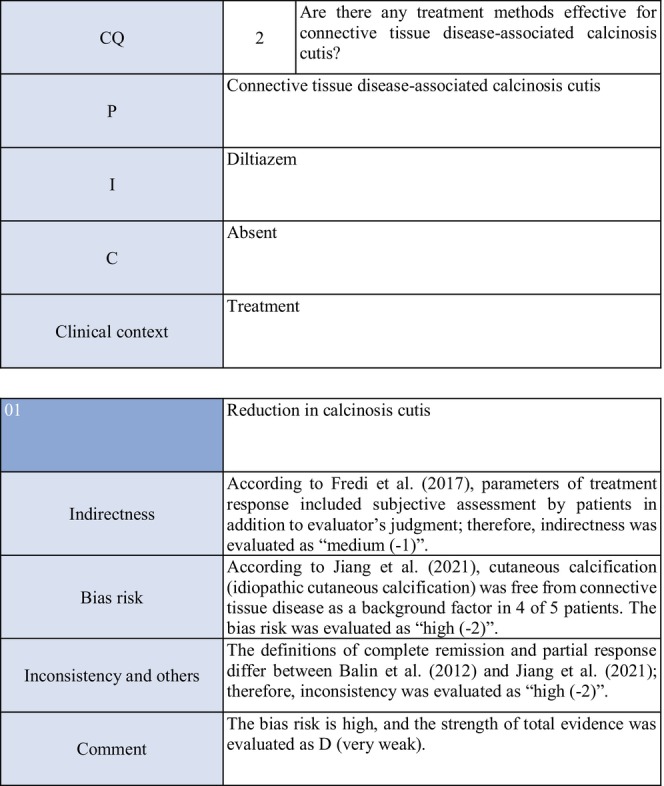
SR‐9: qualitative systematic review. CQ, clinical question.

**FIGURE 15:**
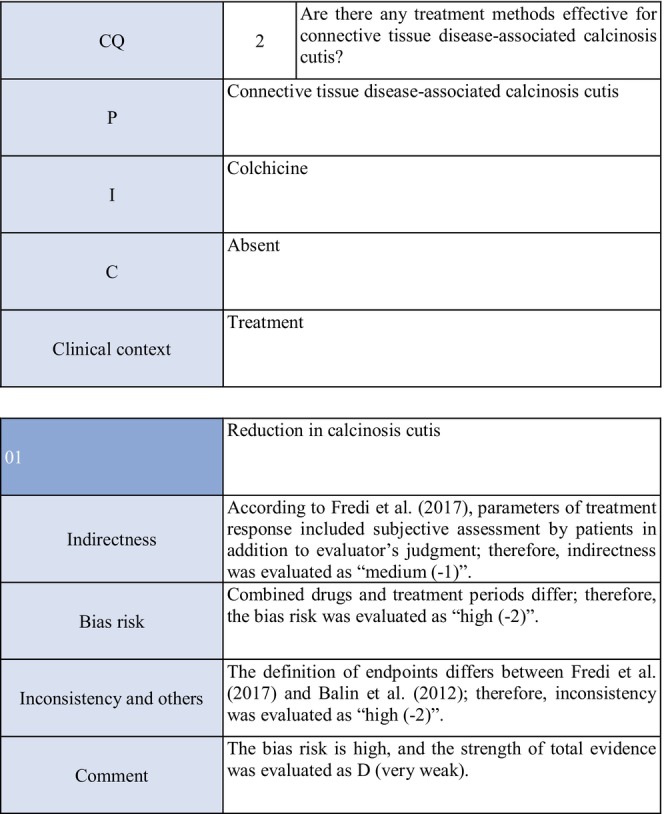
SR‐9: qualitative systematic review. CQ, clinical question.

**FIGURE 16:**
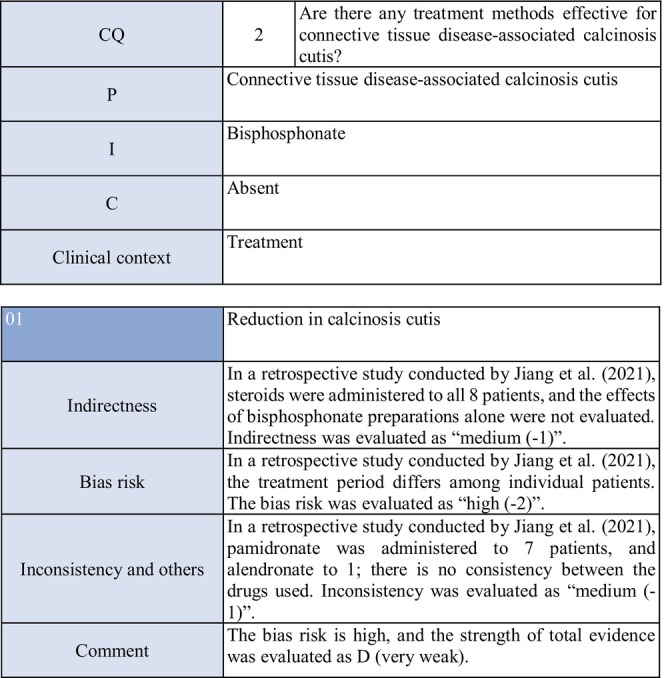
SR‐9: qualitative systematic review. CQ, clinical question.

**FIGURE 17:**
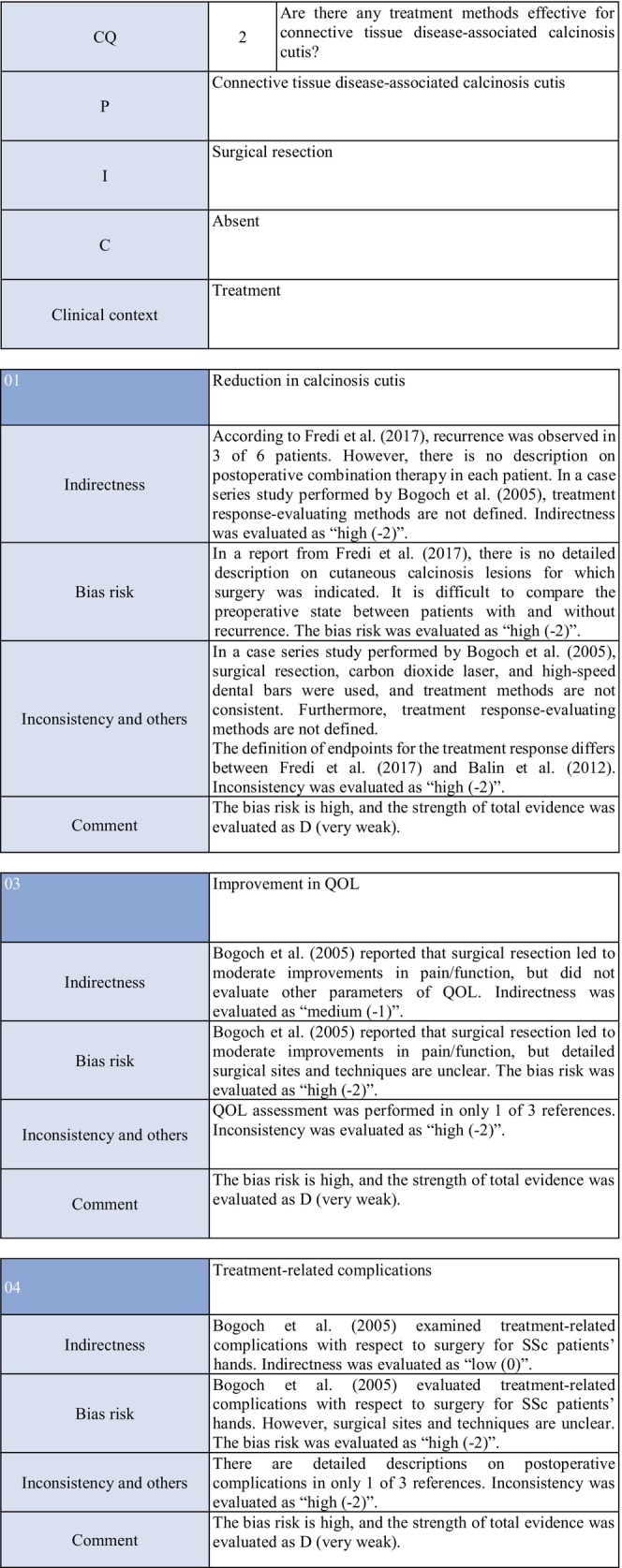
SR‐9: qualitative systematic review. CQ, clinical question; QOL, quality of life.

**FIGURE 18:**
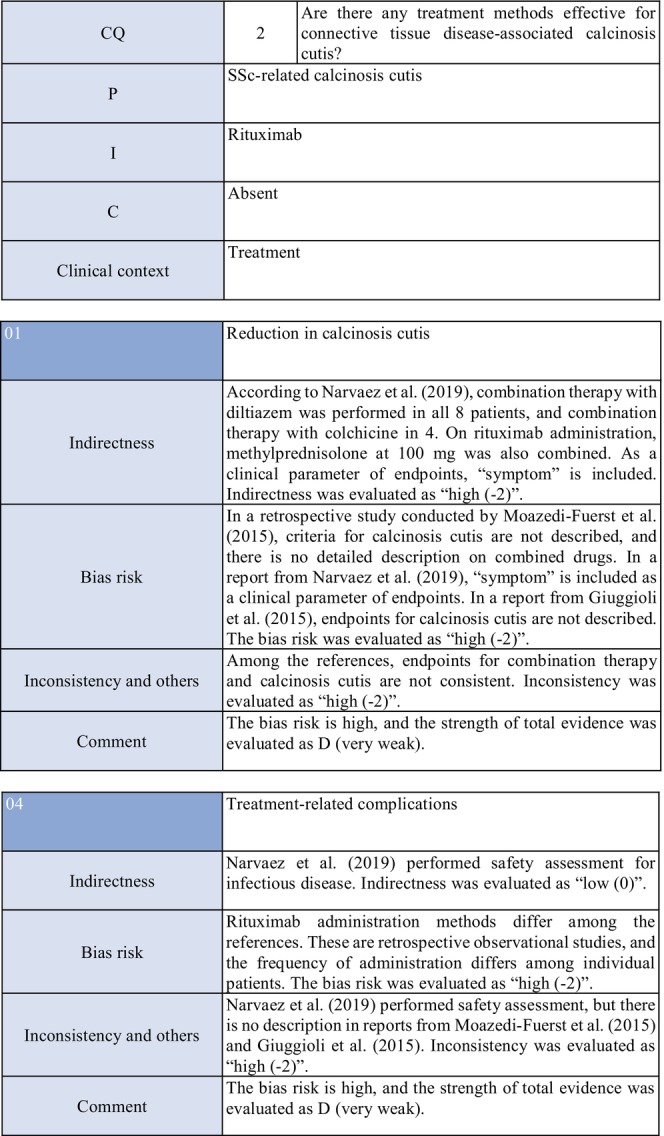
SR‐9: qualitative systematic review. CQ, clinical question; SSc, systemic sclerosis.

**FIGURE 19:**
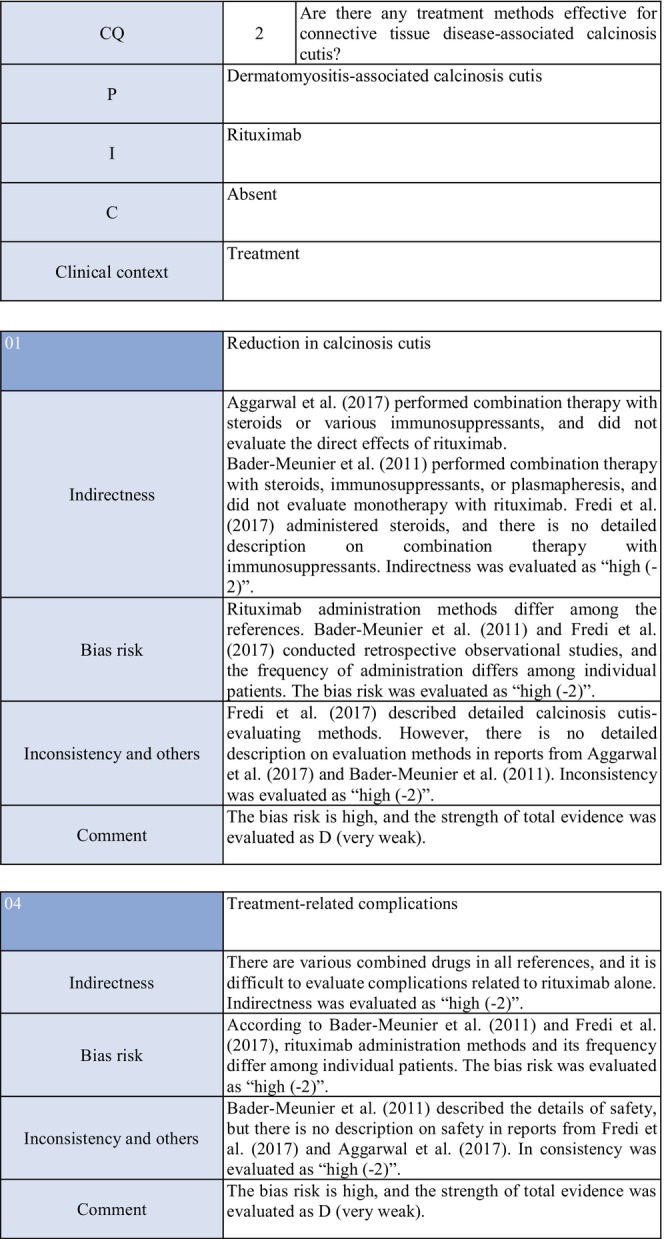
SR‐9: qualitative systematic review. CQ, clinical question.

**FIGURE 20:**
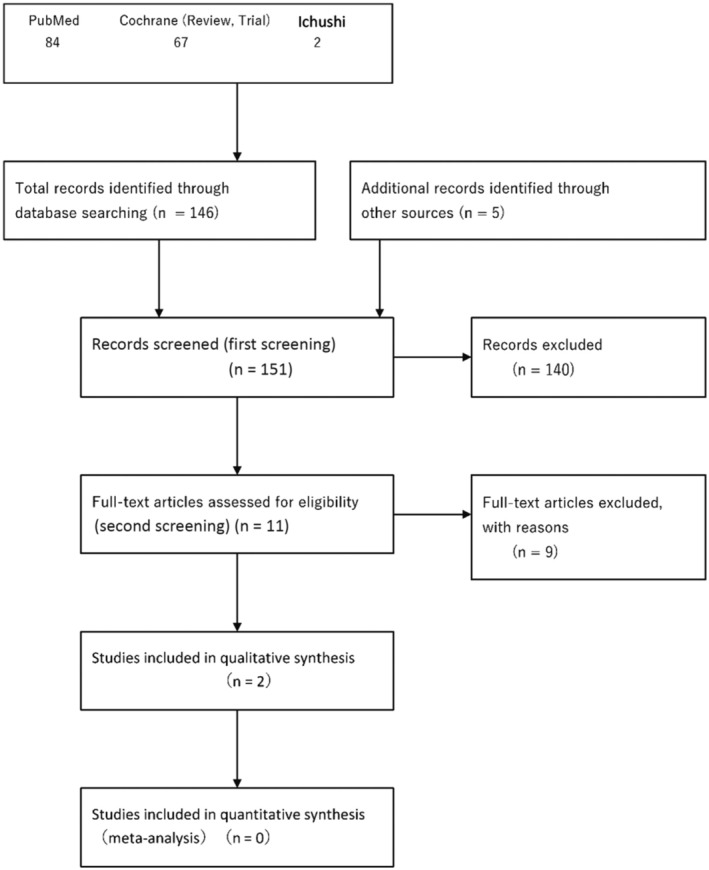
Clinical question 3 (CQ3): flow chart of the literature search.

**FIGURE 21:**
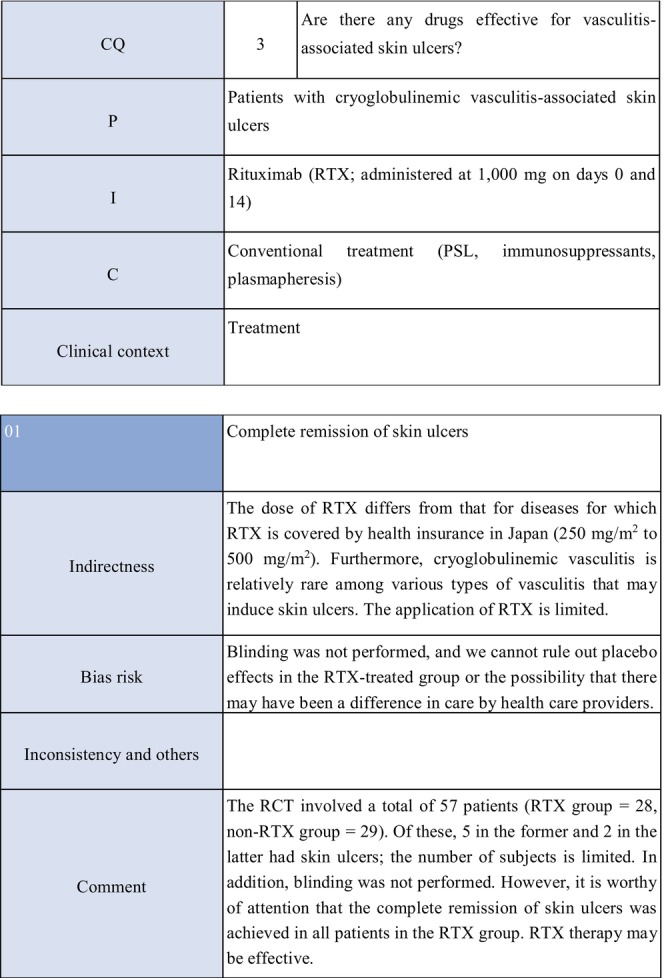
SR‐9: qualitative systematic review. CQ, clinical question; PSL, prednisolone.

**FIGURE 22:**
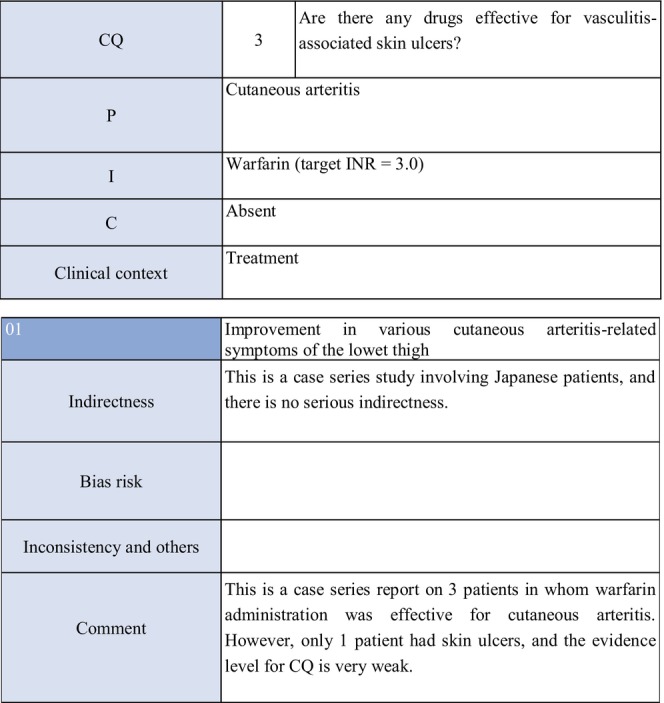
SR‐9: qualitative systematic review. CQ, clinical question; INR, international normalized ratio.

**FIGURE 23:**
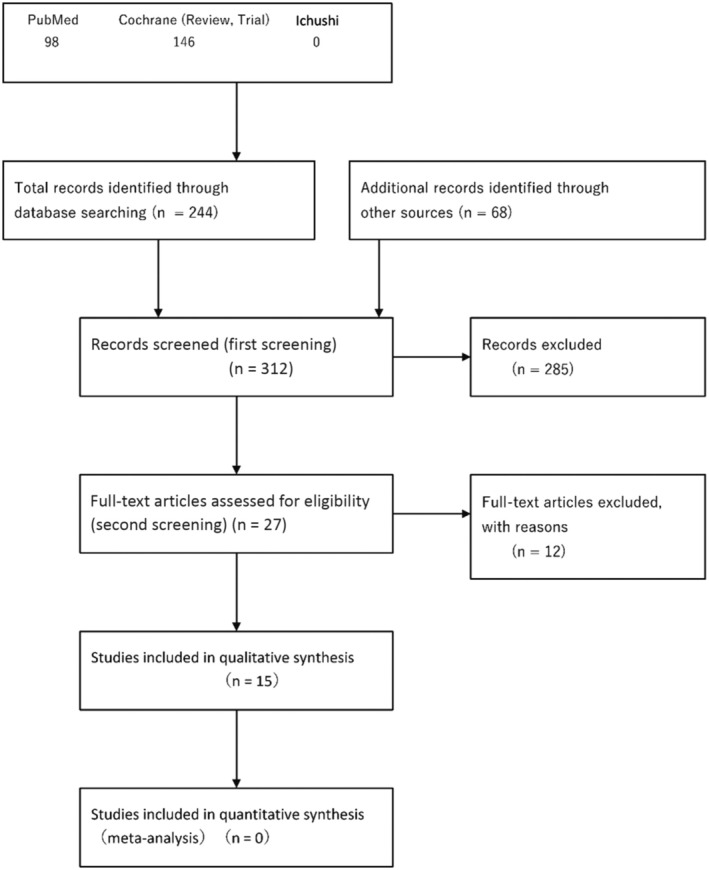
Clinical question 4 (CQ4): flow chart of the literature search.

**FIGURE 24:**
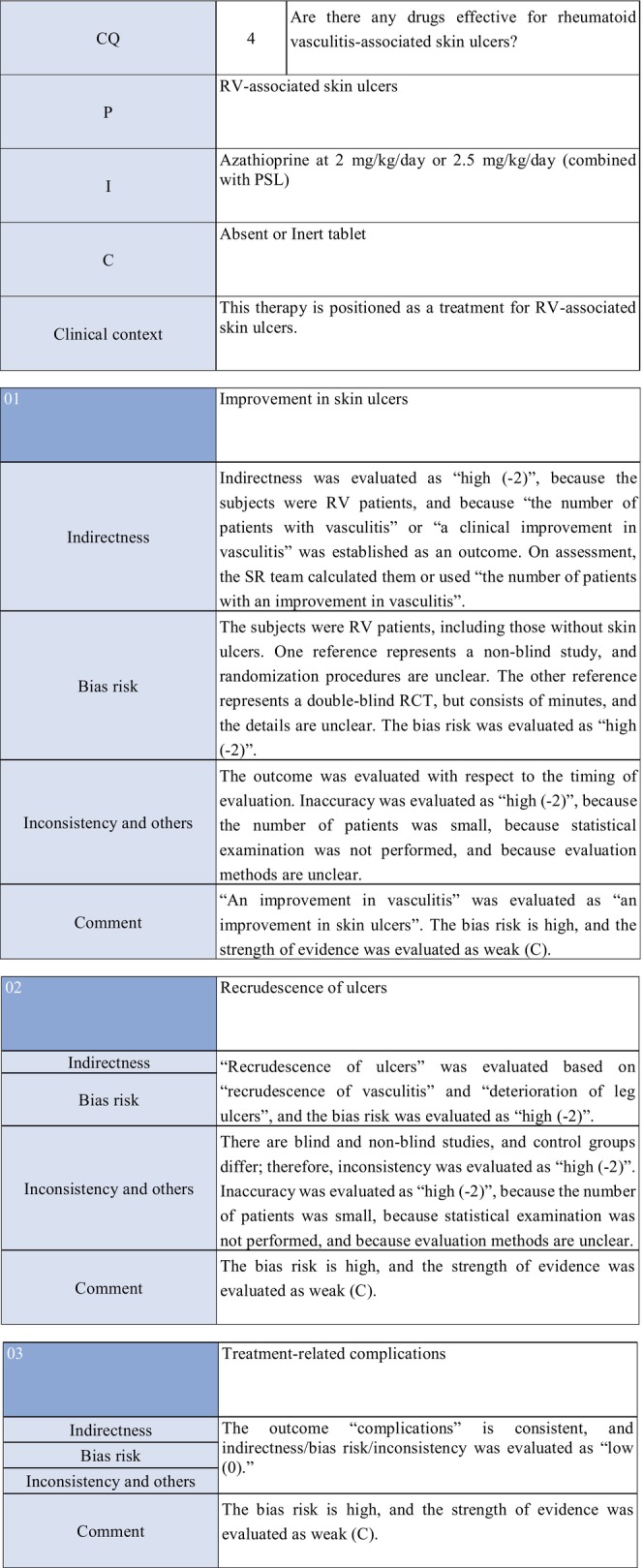
SR‐9: qualitative systematic review. CQ, clinical question; PSL, prednisolone; RCT, randomized controlled trial; RV, rheumatoid vasculitis.

**FIGURE 25:**
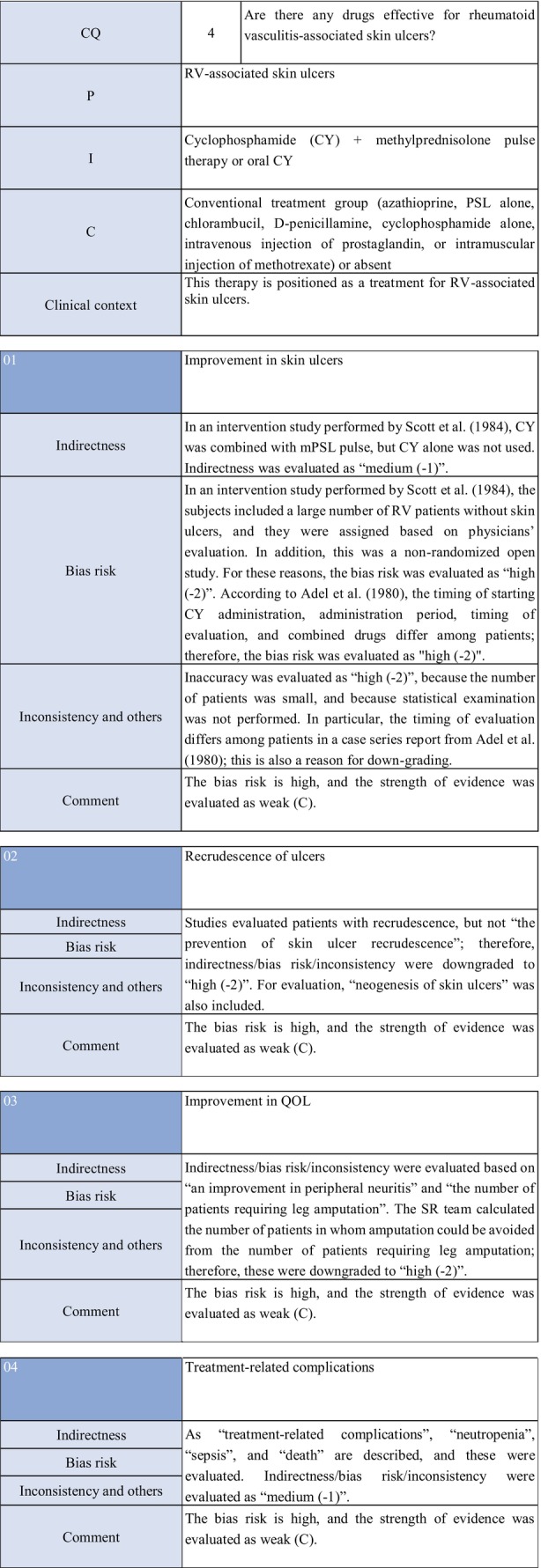
SR‐9: qualitative systematic review. CQ, clinical question; QOL, quality of life; RV, rheumatoid vasculitis.

**FIGURE 26:**
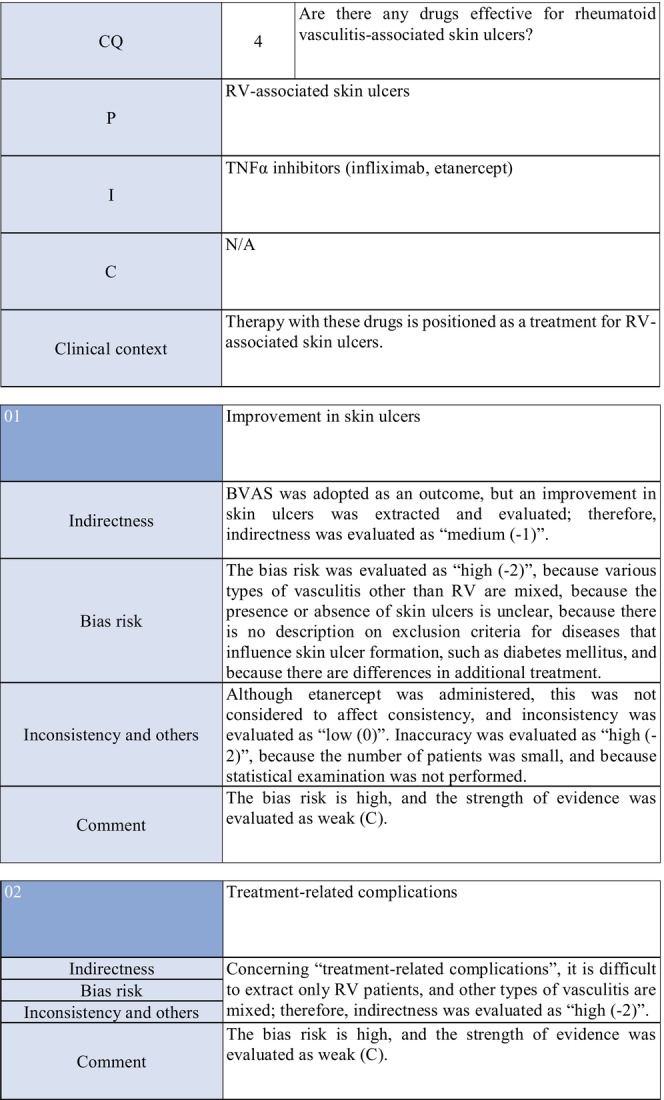
SR‐9: qualitative systematic review. BVAS, Birmingham Vasculitis Activity Score; CQ, clinical question; N/A, not available; RV, rheumatoid vasculitis; TNFα, tumor necrosis factor α; RV, rheumatoid vasculitis.

**FIGURE 27:**
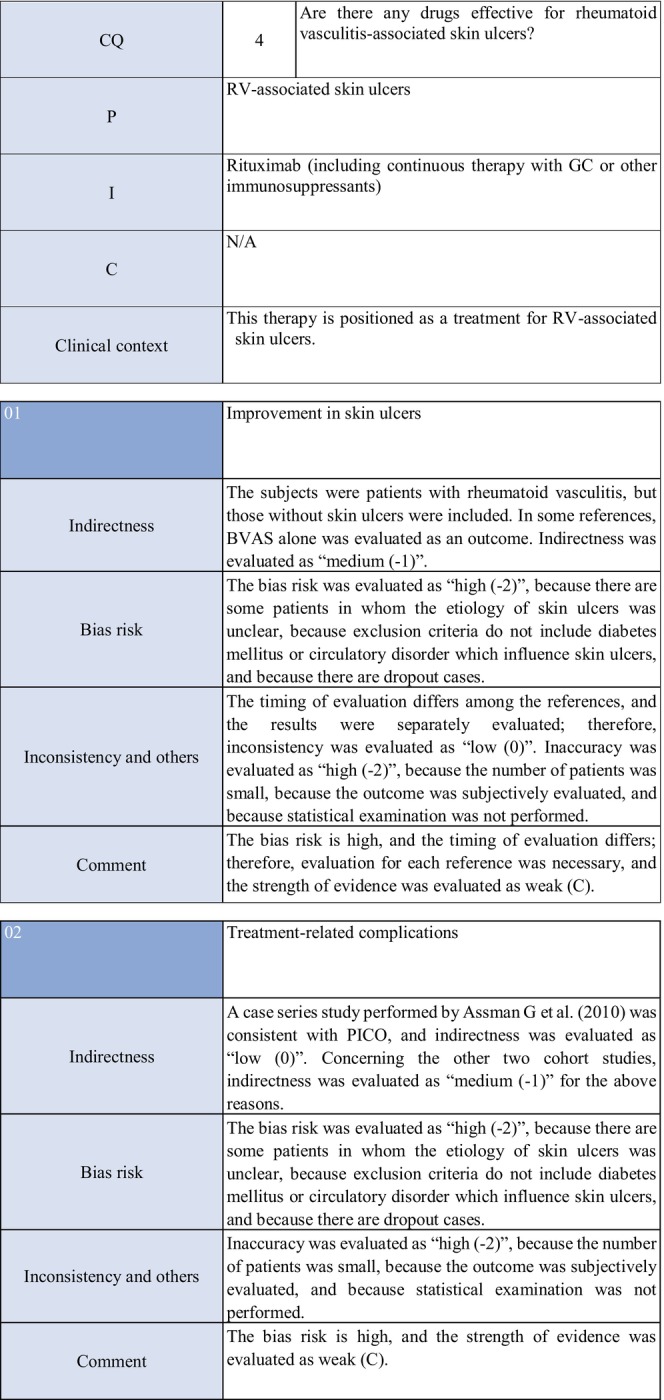
SR‐9: qualitative systematic review. BVAS, Birmingham Vasculitis Activity Score; CQ, clinical question; GC, glucocorticoids; N/A, not available; RV, rheumatoid vasculitis.

**FIGURE 28:**
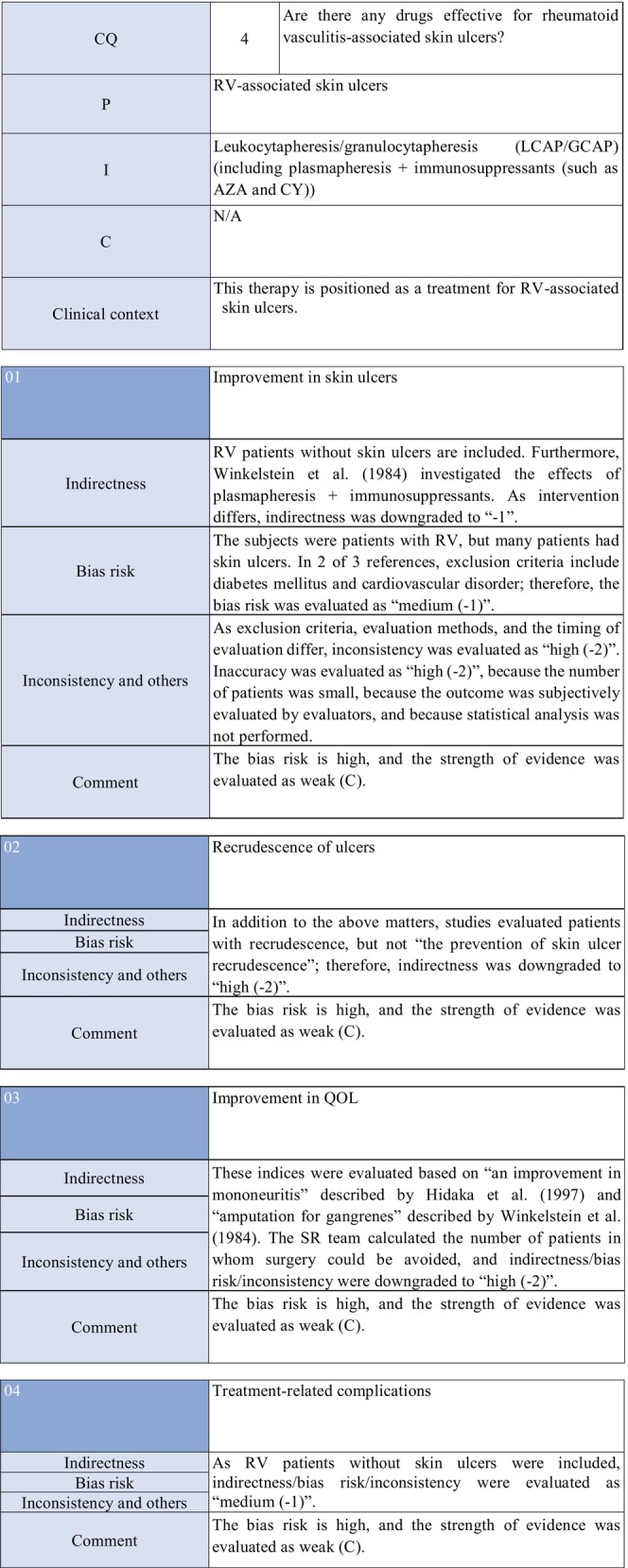
SR‐9: qualitative systematic review. AZA, azathioprine; CY, cyclophosphamide; GCAP, granulocytapheresis; LCAP, leukocytapheresis; N/A, not available; QOL, quality of life; RV, rheumatoid vasculitis.

Based on these results, a summary of the results (SoF) was prepared and presented at a panel meeting.

**CQ2: Are there any treatment methods effective for connective tissue disease–associated calcinosis cutis?**

**Table:** 

Recommendation level	Description of recommendation
Weak recommendation for all treatments	Treatment with warfarin, diltiazem hydrochloride, colchicine, or bisphosphonate preparations or by surgical resection for connective tissue disease–related calcinosis cutis is proposed. Treatment with rituximab for SSc‐related calcinosis cutis is proposed. It is recommended to avoid treatment with rituximab for dermatomyositis‐related calcinosis cutis

### 6.7 Literature search

A request to the Japan Medical Library Association was combined with retrieval by manual search (refer to Supplement Materials with respect to the retrieval style and search results).

Databases used: PubMed, Cochrane Library, Japanese Medical Abstracts Society.

Search period: January 1980 to December 2020.

Results: PubMed: 90 documents, Cochrane Library: 26, and Japanese Medical Abstracts Society: 0 were retrieved. We analyzed 113 documents, excluding database‐duplicated articles.

### 6.8 Outcome

As a result of consideration by the systematic review team, a reduction in calcinosis cutis: importance of outcome 6, inhibition of calcinosis cutis: importance of outcome 5, an improvement in the QOL: importance of outcome 6, and treatment‐related complications: importance of outcome 5 were adopted. The importance of outcome was determined by all guideline‐drafting members' agreement (consistency rate for all imaging procedures: 100%).

### 6.9 Literature screening

Primary screening was performed, and 80 documents describing the above outcomes were selected. Of these, 13 remained through secondary screening. Finally, 13 articles on SSc‐ or dermatomyositis‐related calcinosis remained. A flow chart of literature search is presented (Figure 12).

### 6.10 Evaluation of individual references

With respect to the two RCTs selected on secondary screening, the selection bias, performance bias, detection bias, attribution bias, and other biases were assessed based on the Minds Manual for Guideline Development 2020 version 3.0.

### 6.11 Evaluation of the outcome

In one of the two RCTs extracted this time, the effects of warfarin on connective tissue disease (dermatomyositis, SSc)–related calcinosis were examined. The number of patients was limited: warfarin‐treated group (three) and nonwarfarin‐treated group (four). In the other RCT, the effects of rituximab were investigated in 120 patients with dermatomyositis (72 adults [seven with calcinosis], 48 young patients [22 with calcinosis]). Calcinosis was evaluated as a secondary endpoint, but there was no response. In other references, statistical examination was not performed, and it was impossible to perform a meta‐analysis.

### 6.12 Results

It was difficult to perform a meta‐analysis based on the studies extracted on this screening. When preparing an explanatory text, a broad range of studies, including descriptive research, was introduced, and drugs that may be effective for dermatomyositis‐ and SSc‐related calcinosis were qualitatively evaluated (Figures 13, 14, 15, 16, 17, 18, 19).

Based on these results, a summary of the results (SoF) was prepared, and presented at a panel meeting.

**CQ3: Are there any drugs effective for vasculitis‐associated skin ulcers?**

**Table:** 

Recommendation level	Description of recommendation
Weak recommendation	Systemic steroid administration is proposed because steroids are routinely used as a base drug for vasculitis‐associated skin ulcers in clinical practice and because their effects have been obtained

### 6.13 Literature search

A request to the Japan Medical Library Association was combined with retrieval by manual search (refer to Supplement Materials with respect to the retrieval style and search results).

Databases used: PubMed, Cochrane Library, Japanese Medical Abstracts Society.

Search period: January 1980 to December 2020.

Results: No useful RCT on the treatment of vasculitis‐related skin ulcers was confirmed. When changing some retrieval styles for searching, one RCT of rituximab for cryoglobulinemic vasculitis finally remained. In addition, one case series study of warfarin for cutaneous arteritis was extracted.

### 6.14 Outcome

As a result of consideration by the systematic review team, an improvement in skin ulcers: importance of outcome 6, the prevention of recurrent skin ulcers: importance of outcome 5, an improvement in the QOL: importance of outcome 6, and treatment‐related complications: importance of outcome 5 were adopted. The importance of outcome was determined by all guideline‐drafting members' agreement (consistency rate for all imaging procedures: 100%).

### 6.15 Literature screening

When searching for the treatment of vasculitis‐related skin ulcers, we selected 146 of a total of 153 documents (PubMed [84], Cochrane Library [67], and Ichushi [two]), excluding documents duplicated between PubMed and the Cochrane Library. As articles that were not hit using retrieval styles, but were useful, we extracted a total of five: an RCT of rituximab involving patients with cryoglobulinemic vasculitis, three quoted in our previous guidelines and regarded as candidates for a systematic review, and a new systematic review article on cutaneous arteritis. Of all these articles, a total of 11 were extracted on primary screening, and the contents were reviewed on secondary screening. Finally, one RCT of rituximab for cryoglobulinemic vasculitis and one case series study of warfarin for cutaneous arteritis remained. A retrospective study by Daoud et al. of 79 patients with cutaneous arteritis reported the effects of systemic steroid administration on vasculitis‐related skin ulcers, including 39 with skin ulcers. In addition, one study reported that prednisolone administration at 60 to 80 mg/day was associated with marked improvements in pain, subcutaneous nodules, and skin ulcers, but the actual number of responders is unclear; therefore, this article was not included in the systematic review. A flow chart of literature search is presented in Figure 20.

### 6.16 Evaluation of individual references

For the one RCT selected on secondary screening, the selection bias, performance bias, detection bias, attribution bias, and other biases were assessed based on the Minds Manual for Guideline Development 2020 version 3.0. Blinding was not performed, and we considered that there was a bias. The study examining the effects of warfarin on cutaneous arteritis was a descriptive one, and was excluded on assessment.

### 6.17 Evaluation of the outcome

In the RCT extracted this time, the effects of rituximab on cryoglobulinemic vasculitis were examined, and a total of 57 patients were investigated. Of these, five in the rituximab‐treated group and two in the nonrituximab‐treated group had skin ulcers; the number of patients was limited. This was not considered to be appropriate for quantitative analysis.

### 6.18 Results

It was difficult to perform a meta‐analysis based on the studies extracted on this screening. Furthermore, cryoglobulinemic vasculitis is rare, and the efficacy of drugs for other types of vasculitis was unclear; therefore, when preparing an explanatory text for CQ3, a broad range of studies, including descriptive research, was introduced, and drugs that may be effective for vasculitis‐associated skin ulcers were qualitatively evaluated (Figures 21 and 22).

Based on these results, a summary of the results (SoF) was prepared, and presented at a panel meeting.

**CQ4: Are there any treatment methods effective for rheumatoid vasculitis–associated skin ulcers?**

**Table:** 

Recommendation level	Description of recommendation
Weak recommendation for all drugs	Treatment with azathioprine in combination with steroids or alone, cyclophosphamide + steroid pulse, TNF‐α inhibitors, rituximab, or LCAP/GCAP for rheumatoid vasculitis–related skin ulcers is proposed

### 6.19 Literature search

A request to the Japan Medical Library Association was combined with retrieval by manual search (refer to Supplement Materials with respect to the retrieval style and search results).

Databases used: PubMed, Cochrane Library, Japanese Medical Abstracts Society.

Search period: January 1980 to December 2020.

Results: Literature searching was performed with respect to drugs that are used to treat rheumatoid vasculitis in Japan (steroids, cyclophosphamide + steroid pulse, azathioprine, DDS, rituximab, LCAP/GCAP, biological preparations, methotrexate), and 15 documents examining their usefulness for treating rheumatoid vasculitis with skin ulcers and meeting the PICO (two RCTs, one non‐RCT, four cohort studies, eight case series studies) were adopted.

### 6.20 Outcome

An improvement in skin ulcers: importance of outcome 6, the prevention of recurrent skin ulcers: importance of outcome 5, an improvement in the QOL: importance of outcome 6, and treatment‐related complications: importance of outcome 5 were adopted as outcomes. The importance of outcome was determined by all guideline‐drafting members' agreement (consistency rate for all imaging procedures: 100%).

### 6.21 Literature screening

On primary screening, 312 documents, consisting of 244 obtained by literature searching from the PubMed, Cochrane Library, and Japanese Medical Abstracts Society and 68 obtained by hand searching, were adopted. For hand searching, the literature adopted in the “Guidelines for the management of connective tissue disease/vasculitis‐associated skin ulcers (2017)” by the Japanese Dermatological Association, “Guidelines for Management of Vasculitis Syndrome (JCS 2017),” and MHLW Grants‐in‐Aid for Scientific Research (Intractable Disease Policy Research Project) for Research on “Intractable Vasculitis Guidance for treatment of antiphospholipid antibody syndrome, eosinophilic granulomatosis with polyangiitis, polyarteritis nodosa, and rheumatoid vasculitis 2020” was included. Of these, 27 were adopted for secondary screening. As a result of secondary screening, 15 documents examining the usefulness for treating rheumatoid vasculitis with skin ulcers and meeting the PICO (two RCT, one non‐RCT, six cohort studies, six case series studies) were used for analysis. A flow chart of the literature search is presented in Figure 23.

### 6.22 Evaluation of individual references

No study has limited participants to rheumatoid vasculitis patients with skin ulcers in whom vasculitis was histologically demonstrated. Therefore, there are many studies in which the patients had rheumatoid vasculitis but not skin ulcers, and there are many patients in whom the etiology of skin ulcers was unclear. Many articles with a high bias risk were adopted. Furthermore, there were no studies involving statistical analysis.

### 6.23 Evaluation of the outcome

Of the outcomes adopted: “an improvement in skin ulcers,” “the prevention of recurrent skin ulcers,” “an improvement in the QOL,” and “treatment‐related complications,” no article investigated “the prevention of recurrent skin ulcers,” and recrudescence of ulcers was evaluated; therefore, indirectness was downgraded. “An improvement in the QOL” was reviewed based on the studies establishing an improvement in peripheral neuritis and lower limb amputation as outcomes. The systematic review team reassessed the number of patients in whom lower limb amputation was avoided as an improvement in the QOL, and indirectness was downgraded. No clinical study establishing cost‐effectiveness as an outcome was adopted. The 15 references did not involve statistical examination, and it was impossible to perform a meta‐analysis.

### 6.24 Results

It was difficult to perform a meta‐analysis based on the studies extracted on this screening. When preparing an explanatory text, a broad range of studies, including descriptive research, was introduced, and drugs that may be effective for rheumatoid vasculitis–associated skin ulcers were qualitatively evaluated (Figures 24, 25, 26, 27, 28).

Based on these results, a summary of the results (SoF) was prepared, and presented at a panel meeting.

### 6.25 List of members of the Wound, Pressure Ulcer, and Burn Guidelines Drafting Committee

#### 6.25.1 Supervising committee

Chairperson: Takao TACHIBANA (Hoshigaoka Medical Center).

Vice‐chairperson: Minoru HASEGAWA (University of Fukui), Manabu FUJIMOTO (Osaka University).

Members: Yoshihide ASANO (Tohoku University), Takeshi NAKANISHI (Meiji University of Integrative Medicine), Hiroshi FUJIWARA (Niigata University), Takeo MAEKAWA (Jichi Medical University Saitama Medical Center), Sei‐ichiro MOTEGI (Gunma University), Yuichiro YOSHINO (Japanese Red Cross Kumamoto Hospital).

### 6.26 Drafting committee

#### 6.26.1 Wounds in general

Sei‐ichiro MOTEGI (Gunma University), Masaru ARIMA (Fujita Health University), Toshio ICHIKI (Kyushu University), Ikuko UEDA (Osaka University), Katsuyuki OKADA (Kiryu Kosei General Hospital), Sakae KANEKO (Japanese Red Cross Masuda Hospital), Hiroyuki KANO (Gifu Municipal Hospital), Yuta KURASHIGE (Kurashige Skin Clinic), Akira SHIMIZU (Kanazawa Medical University), Yasuyuki SUMIKAWA (Hokutoukai Sumikawa Dermatology Allergy Clinic), Hidenori TAKAHASHI (Japan Community Health care Organization (JCHO) Fukui Katsuyama General Hospital), Zenshiro TAMAKI (Saitama Prefectural Children's Medical Center), Jun TSUJITA (Social Insurance Inatsuki Hospital), Michio TOKUYAMA (Tokai University), Hideki FUJITA (Nihon University), Koji HABE (Mie University).

#### 6.26.2 Pressure ulcers

Hiroshi FUJIWARA (Niigata University), Ryokichi IRISAWA (Tokyo Medical University), Masaki OTSUKA (Chutoen General Medical Center), Tomoko KAKO (Mie Prefectural General Medical Center), Tatsuya KAJI (Hiroshima City Hiroshima Citizens Hospital), Takafumi KADONO (St. Marianna University School of Medicine), Monji KOGA (Fukuoka University), Kuninori HIROSAKI (Hokkaido Medical Center).

#### 6.26.3 Diabetic skin ulcer/gangrene

Takeshi NAKANISHI (Meiji University of Integrative Medicine), Ryuta IKEGAMI (Ikegami Clinic), Shun OMORI (Kokura Daiichi Hospital), Hiroshi KATO (Nagoya City University), Toshifumi KOMORI (Kyoto Prefectural University of Medicine), Tomomichi SHIMIZU (Tokai University), Kazunari SUGITA (Saga University), Hideaki TANIZAKI (Kansai Medical University), Hideki NAKAJIMA (Kochi University), Shujiro HAYASHI (Dokkyo Medical University), Risa MATSUO (Asahikawa Medical University), Hiroshi MITSUI (University of Yamanashi), Hiroto YANAGISAWA (Saitama Medical University), Michiya YAMAGUCHI (Yamaguchi University), Osamu YAMASAKI (Shimane University).

#### 6.26.4 Connective Tissue Diseases and Vasculitis

Yoshihide ASANO (Tohoku University), Jun ASAI (Kyoto Prefectural University of Medicine), Takayuki ISHII (Toyama Prefectural Central Hospital), Yohei IWATA (Fujita Health University), Akihiko UCHIYAMA (Gunma University), Ken OKAMURA (Yamagata University), Youichi OGAWA (University of Yamanashi), Mari KISHIBE (Asahikawa Medical University), Yuta KOIKE (Nagasaki University), Masanari KODERA (Japan Community Health care Organization (JCHO) Chukyo Hospital), Yorihisa KOTOBUKI (Kotobuki Dermatology Clinic), Noriki FUJIMOTO (Shiga University of Medical Science), Takuya MIYAGI (University of the Ryukyus), Chie MIYABE (Tokyo Women's Medical University), Yukie YAMAGUCHI (Yokohama City University), Ayumi YOSHIZAKI (The University of Tokyo).

#### 6.26.5 Leg Ulcers/Varices

Takeo MAEKAWA (Jichi Medical University Saitama Medical Center), Takeo IDEZUKI (NTT Medical Center Tokyo), Takaaki ITO (Hyogo Medical University), Mayumi OTA (The Jikei University School of Medicine), Hiroshi SAKAI (Osaka University), Yasuko SARAYAMA (Kobe Rosai Hospital), Takamitsu TANAKA (Teikyo University), Hiroyuki NIHARA (Shimane University), Takayuki FUSUMAE (Tokyo Medical Center), Koji MAKINO (National Hospital Organization Kumamoto Medical Center), Hiroshi YATSUSHIRO (Fukui‐ken Saiseikai Hospital).

#### 6.26.6 Burns

Yuichiro YOSHINO (Japanese Red Cross Kumamoto Hospital), Masahiro AMANO (University of Miyazaki), Shiro IINO (University of Fukui), Youichi OMOTO (Omoto Skin Clinic), Masato KAKEDA (Saiseikai Matsusaka General Hospital), Ko KAGOYAMA (University of Toyama), Toru SAITO (Yamagata University), Keisuku SAKAI (National Sanatorium Kikuchi Keifuen), Naotaka DOI (Doi Skin Clinic), Akira HASHIMOTO (Tohoku University), Masahiro HAYASHI (Shin‐Nakamichi Dermatology Clinic), Katsunari MAKINO (Kumamoto University), Naoki MADOKORO (Higashihiroshima Medical Center), Naoya MIKITA (Mikita Dermatology Clinic), Masahito YASUDA (Gunma University), Katsuhiro YAMADA (Akita University).

**COI reporting criteria for participants in the Wound, Pressure Ulcer, Burn Guidelines Supervising and Drafting Committees, participation/nonparticipation criteria, and a list of the COI disclosed** (Prepared in reference to JAMS Guidelines on COI Management in Medical Research (in March 2017, Japanese Association of Medical Sciences, https://jams.med.or.jp/guideline/index.html).

### 6.27 Criteria for judgment of COI disclosure

#### 6.27.1 Participants

1, Presence or absence of officers and advisors in companies and for‐profit organizations and the amounts of their remunerations.

Standard amount: 1 million yen/company/year classification of pension amounts: (1) 1 million yen≤ (2) 5 million yen≤ (3) 10 million yen≤.

2, Ownership of stocks and profits derived from the stocks (profits for the previous year presented in this format).

Standard amount: 1 million yen/company/year classification of pension amounts: (1) 1 million yen≤ (2) 5 million yen≤ (3) 10 million yen≤.

3, Royalty payments for patents by companies and for‐profit organizations.

Standard amount: 1 million yen/company/year classification of pension amounts: (1) 1 million yen≤ (2) 5 million yen≤ (3) 10 million yen≤.

4, Remunerations, such as daily allowances and lecture fees, paid by a company and for‐profit organization for attending a conference (presentations, advice).

Standard amount: 0.5 million yen/company/year classification of pension amounts: (1) 0.5 million yen≤ (2) 1 million yen≤ (3) 2 million yen≤.

5, Fees paid by a company and for‐profit organization for the creation of pamphlets, roundtable discussion articles.

Standard amount: 0.5 million yen/company/year classification of pension amounts: (1) 0.5 million yen≤ (2) 1 million yen≤ (3) 2 million yen≤.

6, Research funds (industry‐academia collaborative research, contract research, clinical trials) provided by a company and for‐profit organization.

Standard amount: 1 million yen/company/year classification of pension amounts: (1) 1 million yen≤ (2) 10 million yen≤ (3) 20 million yen≤.

7, Donations for scholarships and incentives offered by a company and for‐profit organization.

Standard amount: 1 million yen/company/year classification of pension amounts: (1) 1 million yen≤ (2) 5 million yen≤ (3) 10 million yen≤.

8, Endowed courses provided by companies, including those sponsored by companies, with donations of ≥1 million yen.

9, Other remunerations (travel, gifts, not directly related to research).

Standard amount: 50 thousand yen/company/year classification of pension amounts: (1) 50 thousand yen≤ (2) 200 thousand yen≤ (3) 500 thousand yen≤.

#### 6.27.2 Participants' spouses, first‐degree relatives, or those who share income or property interests with participants

1, Presence or absence of officers and advisors in companies and for‐profit organizations and the amounts of their remunerations.

Standard amount: 1 million yen/company/year classification of pension amounts: (1) 1 million yen≤ (2) 5 million yen≤ (3) 10 million yen≤.

2, Ownership of stocks and profits derived from the stocks (profits for the previous year presented in this format).

Standard amount: 1 million yen/company/year classification of pension amounts: (1) 1 million yen≤ (2) 5 million yen≤ (3) 10 million yen≤.

3, Royalty payments for patents by companies and for‐profit organizations.

Standard amount: 1 million yen/company/year classification of pension amounts: (1) 1 million yen≤ (2) 5 million yen≤ (3) 10 million yen≤.

#### 6.27.3 Organizations that participants belong to and those related to departments

1, Research funds (industry‐academia collaborative research, contract research, clinical trials) provided by a company and for‐profit organization.

Standard amount: 10 million yen/company/year classification of pension amounts: (1) 10 million yen≤ (2) 20 million yen≤ (3) 40 million yen≤.

2, Donations for scholarships and incentives offered by a company and for‐profit organization.

Standard amount: 2 million yen/company/year classification of pension amounts: (1) 2 million yen≤ (2) 10 million yen≤ (3) 20 million yen≤.

##### Criteria for exclusion

Members of the guideline drafting committee, their spouses, first‐degree relatives, or those who share income or property interests if they fall under any of the following:

1, Incomes of the officers and advisors of companies and for‐profit organizations (≥1 million yen/company/year);

2, Ownership of stocks and profits generated from the stocks (≥5% of all stocks/company or ≥1 million yen/company/year);

3, Receipt of patent royalties from companies and for‐profit organizations (≥1 million yen/company/year); and

4, Affiliations of endowed courses provided by companies and for‐profit organizations.

#### 6.27.4 Criteria that should be met by the chairperson of the guideline drafting committee

Both individual and organizational COI are classified into Category (1) or below.

#### 6.27.5 Criteria that should be met by the members of the guideline supervisory and drafting committees

Both individual and organizational COI are classified into Category (2) or below. However, the number of persons in Category (2) shall not exceed half of the members of the guideline drafting committee.

#### 6.27.6 List of the COI

Yoshihide ASANO (member of the guideline drafting committee): financial COI AbbVie GK. (Category (2) or below), Eisai Co., Ltd. (Category (2) or below), Kyowa Kirin Co., Ltd. (Category (2) or below), Sanofi K.K. (Category (2) or below), Sun Pharma Japan Ltd. (Category (2) or below), Mitsubishi Tanabe Pharma Corporation (Category (2) or below), Nippon Shinyaku Co., Ltd. (Category (2) or below), Maruho Co., Ltd. (Category (2) or below), Janssen Pharmaceutical K.K. (Category (2) or below).

Jun ASAI (member of the guideline drafting committee): financial COI Sun Pharma Japan Ltd. (Category (2) or below), Maruho Co., Ltd. (Category (2) or below).

Youichi OGAWA (member of the guideline drafting committee): financial COI AbbVie GK. (Category (2) or below), Sanofi K.K. (Category (2) or below), Janssen Pharmaceutical K.K. (Category (2) or below).

Yuta KOIKE (member of the guideline drafting committee): financial COI AbbVie GK. (Category (2) or below), Eli Lilly Japan K.K. (Category (2) or below), Maruho Co., Ltd. (Category (2) or below).

Yorihisa KOTOBUKI (member of the guideline drafting committee): financial COI Sun Pharma Japan Ltd. (Category (2) or below), Maruho Co., Ltd. (Category (2) or below).

Noriki FUJIMOTO (member of the guideline drafting committee): financial COI Sun Pharma Japan Ltd. (Category (2) or below).

Takuya MIYAGI (member of the guideline drafting committee): financial COI AbbVie Inc. (Category (2) or below), Eisai Co., Ltd. (Category (2) or below), Kyowa Kirin Co., Ltd. (Category (2) or below), Sun Pharma Japan Ltd. (Category (2) or below), Eli Lilly Japan K.K. (Category (2) or below), Maruho Co., Ltd. (Category (2) or below), Janssen Pharmaceutical K.K. (Category (2) or below).

Yukie YAMAGUCHI (member of the guideline drafting committee): financial COI AbbVie GK. (Category (2) or below), Kyowa Kirin Co., Ltd. (Category (2) or below), Sanofi K.K. (Category (2) or below), Sun Pharma Japan Ltd. (Category (2) or below), Maruho Co., Ltd. (Category (2) or below), UCB Japan Co. Ltd. (Category (2) or below).

Minoru HASEGAWA (member of the guideline supervising committee), financial COI Ono Pharmaceutical Co., Ltd. (Category (2) or below), Maruho Co., Ltd. (Category (2) or below).

Manabu FUJIMOTO (member of the guideline supervising committee), financial COI Maruho Co., Ltd. (Category (2) or below), Janssen Pharmaceutical K.K. (Category (2) or below).

#### 6.27.7 Nonfinancial COI

Takafumi Kadono is the Editor‐in‐Chief of the *Journal of Dermatology* and a co‐author of this article. Dr Kadono is excluded from editorial decision‐making related to the acceptance and publication of this article.

Minoru Hasegawa, Mari Kishibe, and Hideki Fujita are Editorial Board members of the *Journal of Dermatology*. Minoru Hasegawa and Mari Kishibe are co‐authors of this article, and Hideki Fujita is a member of the drafting committee of Wounds and General. To minimize bias, they were excluded from all editorial decision‐making related to the acceptance of this article for publication.
